# Development of a Multi-Epitope Vaccine for *Mycoplasma hyopneumoniae* and Evaluation of Its Immune Responses in Mice and Piglets

**DOI:** 10.3390/ijms23147899

**Published:** 2022-07-18

**Authors:** Gaojian Li, Jinqi Shu, Jing Jin, Jianhong Shu, Huapeng Feng, Jian Chen, Yulong He

**Affiliations:** 1 Department of Biopharmacy, College of Life Sciences and Medicine, Zhejiang Sci-Tech University, Hangzhou 310018, China; 201820201018@mails.zstu.edu.cn (G.L.); 201920201058@mails.zstu.edu.cn (J.S.); 202020801037@mails.zstu.edu.cn (J.J.); shujianhong@zstu.edu.cn (J.S.); fenghuapeng@zstu.edu.cn (H.F.); chj1999@zstu.edu.cn (J.C.); 2 Shaoxing Biomedical Research Institute, Zhejiang Sci-Tech University, Shaoxing 312000, China

**Keywords:** *Mycoplasma hyopneumoniae*, reverse vaccinology, multi-epitope vaccine design, immunization of mice and piglets, immune response evaluation

## Abstract

*Mycoplasma hyopneumoniae* (Mhp), the primary pathogen causing *Mycoplasma* pneumonia of swine (MPS), brings massive economic losses worldwide. Genomic variability and post-translational protein modification can enhance the immune evasion of Mhp, which makes MPS prone to recurrent outbreaks on farms, even with vaccination or other treatments. The reverse vaccinology pipeline has been developed as an attractive potential method for vaccine development due to its high efficiency and applicability. In this study, a multi-epitope vaccine for Mhp was developed, and its immune responses were evaluated in mice and piglets. Genomic core proteins of Mhp were retrieved through pan-genome analysis, and four immunodominant antigens were screened by host homologous protein removal, membrane protein screening, and virulence factor identification. One immunodominant antigen, AAV27984.1 (membrane nuclease), was expressed by *E. coli* and named rMhp597. For epitope prioritization, 35 B-cell-derived epitopes were identified from the four immunodominant antigens, and 10 MHC-I and 6 MHC-II binding epitopes were further identified. The MHC-I/II binding epitopes were merged and combined to produce recombinant proteins MhpMEV and MhpMEVC6His, which were used for animal immunization and structural analysis, respectively. Immunization of mice and piglets demonstrated that MhpMEV could induce humoral and cellular immune responses. The mouse serum antibodies could detect all 11 synthetic epitopes, and the piglet antiserum suppressed the nuclease activity of rMhp597. Moreover, piglet serum antibodies could also detect cultured Mhp strain 168. In summary, this study provides immunoassay results for a multi-epitope vaccine derived from the reverse vaccinology pipeline, and offers an alternative vaccine for MPS.

## 1. Introduction

*Mycoplasma hyopneumoniae* (Mhp), the primary pathogen causing MPS [1], brings massive economic losses worldwide. MPS can cause decreased daily weight gain and severe clinical symptoms in growing and finishing pigs, leading to secondary infections with other pathogens [2]. Commercial vaccines to prevent MPS mainly include attenuated or inactivated vaccines, supplemented with adjuvants [3], and Mhp infections are prone to recurrent outbreaks in farms, indicating that these vaccines cannot completely eliminate Mhp from farms or bodies [4]. We proposed earlier that genomic variability and post-translational protein modification enhanced the immune evasion of Mhp [5]. As shown in Supplementary File S1, we performed phylogenetic tree construction for some reported virulence factors of Mhp and presented multiple sequence alignment results of the repeat segments of P146 and P97. P146 and P97 are usually used as molecules for Mhp genome classification [6,7,8] and vaccine development, while the protein variety in different strains may decrease the universal applicability of vaccines. These results suggest significant differences between the same types of virulence factors from different Mhp strains, which challenges the general applicability of vaccines based on these virulence factors. More importantly, in vitro culture of Mhp is difficult due to its complex medium requirements [9], which limits clinical studies and adversely affects vaccine development. 

With the development of computational immunology and vaccine informatics, a reverse vaccinology pipeline has been established [10], and some multi-epitope vaccines have been reported [11,12,13,14,15]. These vaccines are free from allergenic, toxic, or unwanted peptide fragments, which improves their safety. The reverse vaccinology pipeline has some attractive features compared to traditional approaches to vaccine development. Cultivation of target pathogens in vitro is unnecessary, and all proteins produced by the pathogens can be used as candidate antigens. In addition, this pipeline is an effective time-saver, and a vaccine candidate was reported in the early stages of the coronavirus disease 2019 (COVID-19) outbreak [16]. On the other hand, molecular docking can validate the binding efficiency of designed vaccines to immune receptors (such as Toll-like receptors), and advances in protein structure prediction have made this approach more convenient [17]. The main objective of the reverse vaccinology pipeline is to highlight immunodominant and antigenic epitopes that could induce effective and safe immune responses, and then meet vaccine candidate requirements [18]. Supplementary File S2 lists the database, software, and web services used in this study. 

**Figure 1:**
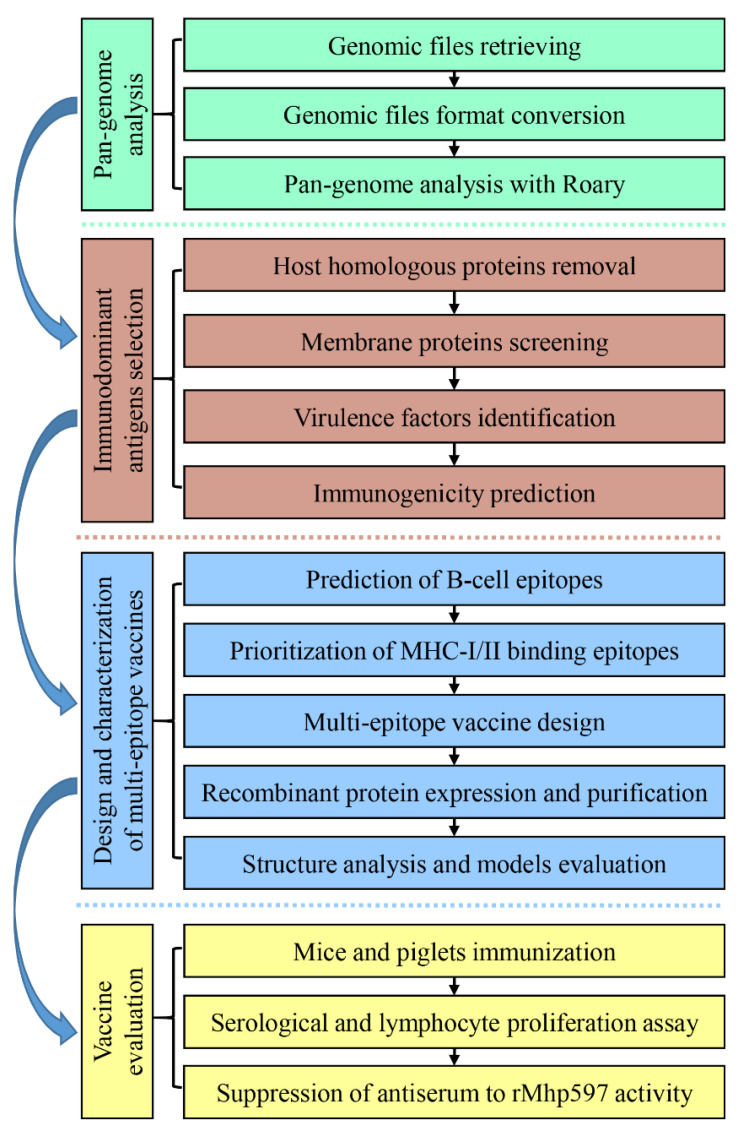
Pipelines for reverse vaccinology and animal immune experiments in this study. Pan-genome analysis was used first to identify the conservative core proteins. Immunodominant antigens were selected by host homologous protein removal, membrane protein selection, and virulence factor database alignment. MHC-I/II binding epitopes were screened by different web services and finally integrated to produce the artificial proteins MhpMEV and MhpMEVC6His. The immunogenicity of MhpMEV was evaluated in animal experiments, and MhpMEVC6His was used for structure analysis.

In this study, we developed a multi-epitope vaccine for MPS, MhpMEV, based on the reverse vaccinology pipeline. The secondary structure of MhpMEV was determined by circular dichroism, and the three-dimensional structure was predicted and validated in silico. Moreover, the immunogenicity of MhpMEV was evaluated in mice and piglets, indicating that it could induce humoral and cellular immune responses. Figure 1 presents the pipeline of this research. Briefly, this study provides wet-lab immunoassay results for a multi-epitope vaccine derived from the reverse vaccinology pipeline and also provides a conceptual multi-epitope vaccine for MPS. 

## 2. Results

### 2.1. Four Immunodominant Antigens Were Screened by Pan-Genome Analysis and Further Selection

**Figure 2:**
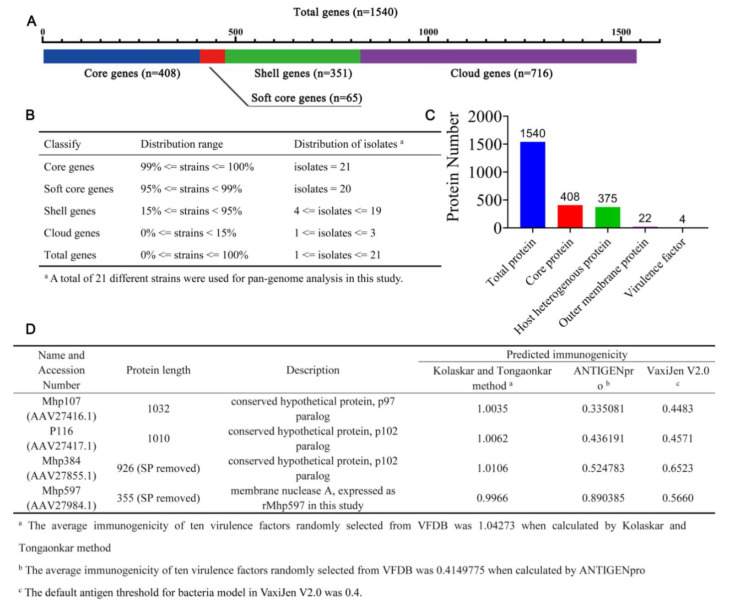
Pan-genome analysis of twenty-one Mhp strains and the immunodominant antigen selection. (**A**,**B**) Summary statistics of Mhp pan-genome analysis results; 408 core genes were identified and mapped to Mhp strain 232 genome to find the core proteins. (**C**) Changes in protein quantity during the screening processes included pan-genome analysis, host homologous protein removal, subcellular localization, and virulence factor database alignment. (**D**) The immunogenicity of the four immunodominant antigens was calculated by Kolaskar and Tongaonkar method, ANTIGENpro, and VaxiJen V2.0.

For pan-genome analysis, Roary was installed in a Bio-Linux 8 system, and the dependency check is shown in Supplementary File S2 Figure S3. By pan-genome analysis, 408 conservative core genes were identified, and the summary statistics are shown in Figure 2A,B. Then, the core genes were mapped to the Mhp strain 232 genome to identify the core proteins. The immunodominant antigens were screened by host homologous protein removal, subcellular localization, and sequence alignment against the virulence factor database. Changes in protein quantity during the screening processes are shown in Figure 2C, and the proteins are listed in Supplementary File S3. Finally, 22 outer membrane proteins were identified among the 375 host heterogeneous proteins, and four immunodominant antigens were identified. All of the immunodominant antigens had been reported as functional virulence factors, including three adhesins, AAV27416.1 [19], AAV27417.1 [20], and AAV27855.1 [21], and one membrane nuclease, AAV27984.1 [22]. The results of the expression and purification of AAV27984.1 are provided in Supplementary File S4. The purified protein was named rMhp597, and its nuclease activity was also confirmed. Through protein characterization analysis, each immunodominant antigen was shown to have a single trans-membrane helix; only AAV27855.1 and AAV27984.1 had signal peptide cleavage sites and were removed in further epitope selection. Figure 2D shows the immunogenicity of each antigen, calculated by the Kolaskar and Tongaonkar method, ANTIGENpro, and VaxiJen V2.0. 

### 2.2. Thirty-Five B-Cell Epitopes Were Identified within the Immunodominant Antigens 

ABCPred found 312 potential B-cell epitopes with default parameters within the four immunodominant antigens, and Bcepred screened 282 linear B-cell epitopes based on physical and chemical properties. Then, the BLASTp program was performed to find the epitopes that were co-identified by the two services. Finally, 35 non-redundant B-cell epitopes were identified, each 16 amino acids in length. The screening processes are presented in Supplementary File S5. 

### 2.3. Ten MHC-II Binding Epitopes Were Identified within B-Cell Epitopes

EpiTOP 3.0 was used to search for MHC-II binding epitopes within the 35 non-redundant B-cell epitopes and 11 epitopes were identified that could bind to more than three MHC II alleles. The antigenicity of the 11 epitopes was calculated by VaxiJen V2.0, and epitopes DSEIQKLKKPELNSLE and TFFLDGKSSFSKSDNK were removed, as their antigenic potential was less than 0.5. Through the prediction of MHCPred server version 2.0, 58 sub-epitopes were screened that could bind to predominant HLA-II allele DRB1*0101 (IC50 < 500 nM), and the length of these epitopes was split into nine amino acids. Then, the 58 epitopes were submitted to VirulentPred to determine their cytotoxicity, and all of them were positive. IFNepitope predicted the ability to induce IFN-γ production and identified 10 epitopes, which were regarded as MHC-II binding epitopes. The results of the selection process of MHC-II binding epitopes are shown in Supplementary File S5.

### 2.4. Six MHC-I Binding Epitopes Were Screened within the Four Immunodominant Antigens

The MHC-I binding prediction tool v2.23 found 43 potential epitopes within the four immunodominant antigens, which could bind to porcine MHC-I alleles with IC50 < 500 nM. The length of each epitope was split into nine amino acids, but some epitopes contained overlapping sequences. Then, their immunogenicity was calculated by the MHC I immunogenicity score, and 22 candidate epitopes with a score >0 were selected. To further improve the accuracy, the obtained epitopes were submitted to MHCPred server version 2.0 to calculate their binding efficiency with the predominant HLA-A*0201, and eight potential epitopes were identified. Finally, VaxiJen V2.0 prioritized the MHC-I binding epitopes and identified six epitopes as “possible antigens”, which were regarded as MHC-I binding epitopes. The results of the selection process of MHC-I binding epitopes are shown in Supplementary File S5.

### 2.5. MhpMEV Was Designed by Epitope Combinations and Expressed in E. coli

**Figure 3:**
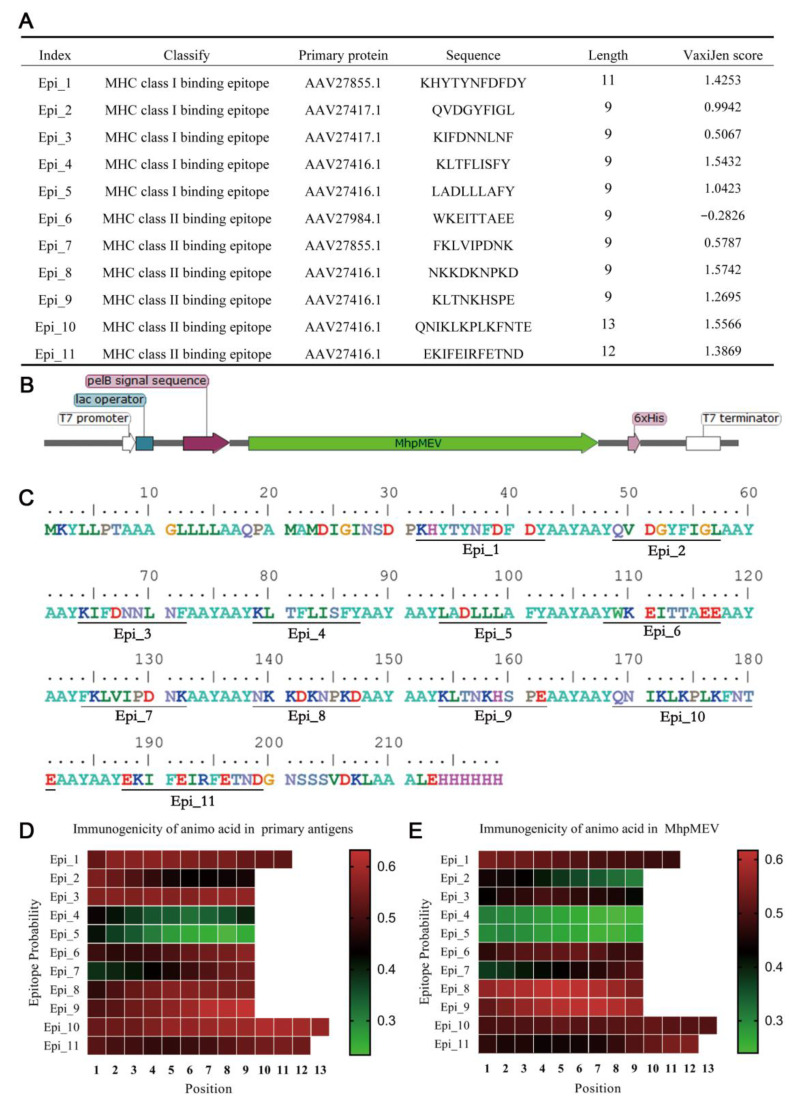
The design of the multi-epitope vaccine. (**A**) Identified MHC-I/II binding epitopes and their immunogenicity calculated by VaxiJen V2.0. (**B**) The open reading frame features of the MhpMEV expression vector. (**C**) The protein sequence of MhpMEV and the different epitopes are underlined. The immunogenicity of each epitope amino acid calculated by BepiPred 2.0 in the immunodominant antigens and MhpMEV is presented in (**D**,**E**), respectively.

MHC-I/II binding epitopes that contained overlapping sequences were merged and finally 11 non-redundant MHC-I/II binding epitopes were obtained. These epitopes are shown in Figure 3A. The immunogenicity was calculated by VaxiJen V2.0. The MHC-I/II binding epitopes were combined by the AAYAAY linker to produce an integrated protein named MhpMEV. The gene of MhpMEV was codon-optimized and synthesized by GenScript, and then it was inserted into the pET22b plasmid, which retained the 6×His Tag at its C-terminus. This recombinant plasmid was named pET22b-MhpMEV and transformed into *E. coli* BL21(DE3) competent cells for protein expression. The open reading frame features and MhpMEV protein sequence are presented in Figure 3B,C. The immunogenicity of amino acids calculated by BepiPred 2.0 in immunodominant antigens and MhpMEV is presented in Figure 3D,E.

### 2.6. MhpMEV Was Expressed as Inclusion Bodies and Refolded by Gradient Dialysis

**Figure 4:**
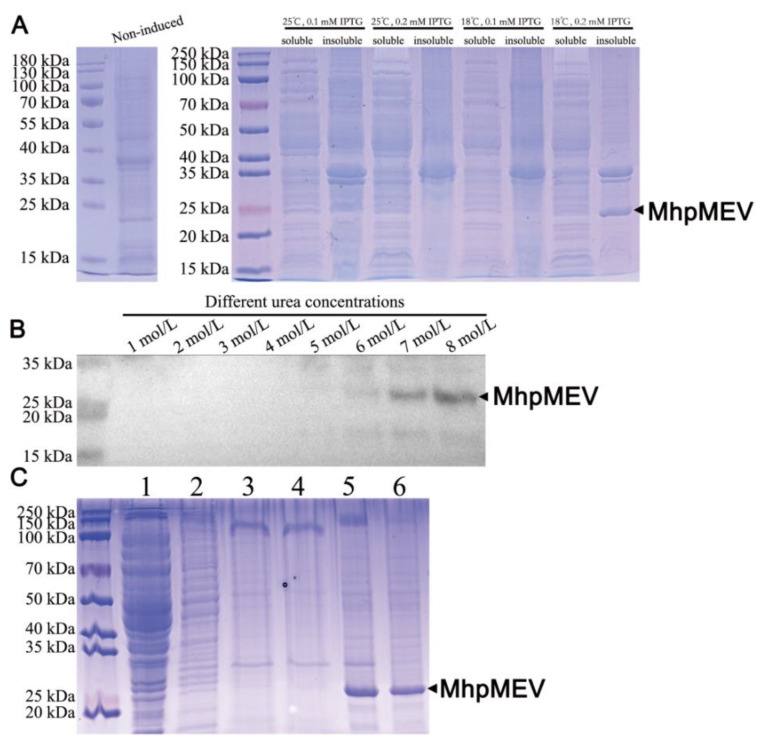
Protein expression and inclusion body refolding. (**A**) The expression results of MhpMEV in different induction conditions (Right); the non-induced cells were used as negative control (Left). The theoretical molecular weight of MhpMEV was 24.5 kDa, and the specific band could be obtained when incubated with 0.2 mM IPTG at 18 °C. (**B**) Western blotting analysis of MhpMEV with mouse monoclonal anti-His antibody. (**C**) SDS-PAGE analysis of the inclusion body washing and refolding. Lane 1: supernatant liquor after bacterial cell disruption; Lane 2: WB-I eluate; Lane 3: WB-II eluate; Lane 4: WB-I III eluate; Lane 5: 8 mol/L urea dissolving solution; Lane 6: supernatant liquor after refolding.

Four induction conditions were explored for MhpMEV expression, and the results are shown in Figure 4A. The theoretical molecular weight of MhpMEV was 24.5 kDa, and the specific band could be obtained with 0.2 mM IPTG and incubated at 18 °C. MhpMEV was expressed as inclusion bodies, and the Western blotting result is presented in Figure 4B. The inclusion bodies were washed three times with WB-I, WB-II, and WB-III washing buffers, with Triton X-100 and 0.3 or 0.6 mol/L urea added as detergents to remove impurities. Then, the inclusion bodies were dissolved in 8 mol/L urea and refolded by gradient dialysis in RB-I, RB-II, RB-III, and RB-IV refolding buffers. The SDS-PAGE results of the washing and refolding processes are shown in Figure 4C. MhpMEV was concentrated by ultrafiltration to 1 mg/mL and stored at −80 °C for animal immunization. 

### 2.7. Secondary Structural Analysis 

**Figure 5:**
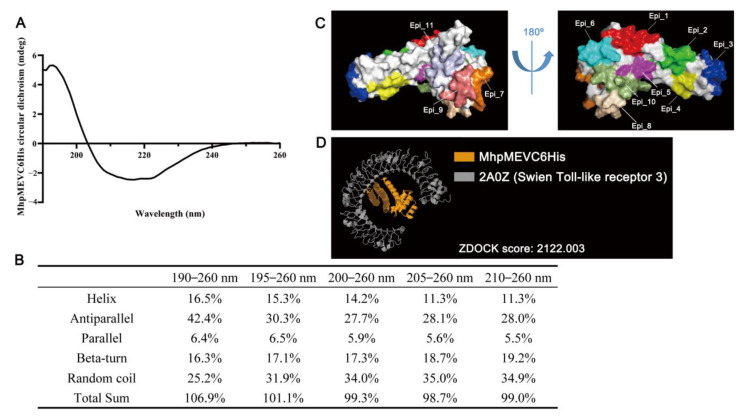
Structure analysis and molecular docking of MhpMEVC6His (**A**) The far-UV circular dichroism spectra of MhpMEVC6His. (**B**) The ratio of different secondary structures calculated by CDNN in MhpMEVC6His. (**C**) The three-dimensional structure model of MhpMEVC6His was predicted by Robetta and the 11 epitopes are colored and demonstrated. (**D**) Molecular docking result of MhpMEVC6His and swine Toll-like receptor 3; this model presented the best binding conformation with the highest ZDOCK score.

MhpMEV was redesigned as MhpMEVC6His for further purification, which added a 6×His tag to its C-terminus directly (Supplementary File S6). The secondary structure of MhpMEVC6His was identified by circular dichroism and is presented in Figure 5A. The ratios of the secondary structures calculated by CDNN are listed in Figure 5B, and the results indicate that random coils and antiparallels/parallels account for the largest proportion. RaptorX Property analysis showed that MhpMEVC6His had 61% alpha-helix and 38% coil. The three-state secondary structure, relevant solvent accessibility, and disordered regions are given in Supplementary File S6, and they demonstrated significant differences when compared with the calculation results of circular dichroism. 

### 2.8. MhpMEVC6His Three-Dimensional Structure Prediction and Molecular Docking

Robetta, I-TASSER, and AlphaFold were used to predict the three-dimensional structure of MhpMEVC6His, and the results are shown in Supplementary File S6. ProSA Web determined the Z-scores of the models, and all Z-scores were within the range of values typically found for native proteins of similar sizes. By Ramachandran plot analysis, most of the residues in the Robetta model were found in favored (93.8%) or allowed (6.2%) regions, while, in the I-TASSER and AlphaFold models, 81.2 and 84.9% were found in favored regions. In Ramachandran plot analysis, based on an analysis of 118 structures with a resolution of at least 2.0 Angstroms and R-factor no greater than 20%, a good-quality model would be expected to have over 90% in the most favored regions. The validation results indicate that the Robetta model was better than the I-TASSER and AlphaFold models for artificial protein MhpMEVC6His. The Robetta model is presented in Figure 5C, with the 11 epitopes indicated by different colors. 

The molecular model of MhpMEVC6His predicted by Robetta was docked with swine immune-associated receptors through ZDOCK, including MHC class I antigens (3QQ4, 5YLX, 6A6H, and 7EMA) and Toll-like receptor 3 (2A0Z). The top 10 binding models with the highest ZDOCK scores for each receptor are listed in Supplementary File S6. Among these models, Toll-like receptor 3 had the highest ZDOCK score, as shown in Figure 5D. On the other hand, MhpMEVC6His could also bind to the other receptors, with similar or slightly lower ZDOCK scores. The molecular docking results indicate that MhpMEVC6His could induce immune responses in swine, which were evaluated in the following animal immunization.

### 2.9. Mouse Immunization

**Figure 6:**
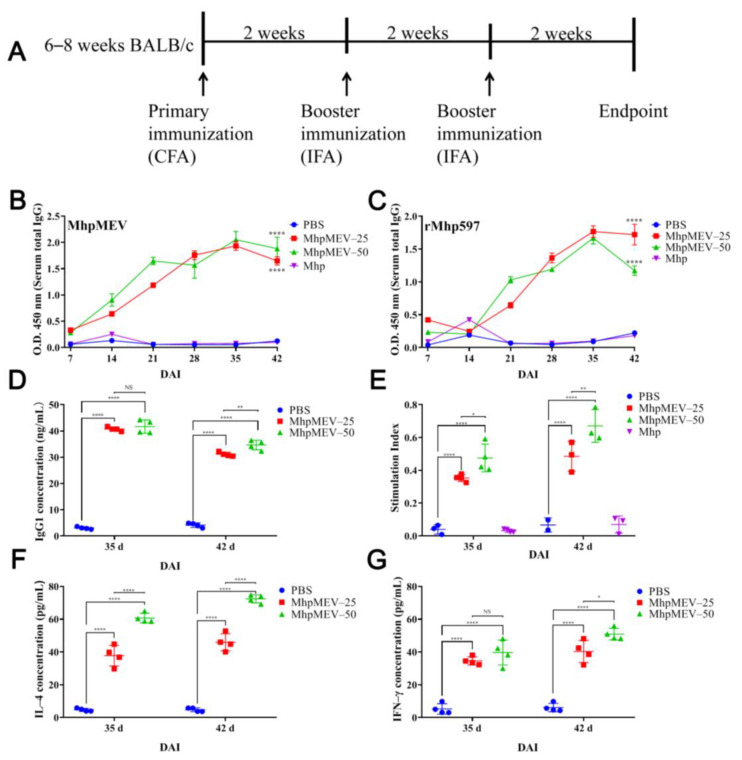
Immunoassay results of mouse immune experiment. (**A**) The vaccination schedule of the mouse immune experiment. CFA: complete Freund’s adjuvant, IFA: incomplete Freund’s adjuvant. (**B**) Serum ELISA results against MhpMEV. (**C**) Serum ELISA results against rMhp597. The symbol **** indicates a statistical difference between the MhpMEV-25 or MhpMEV-50 groups and the PBS group in the figures. (**D**) Serum total IgG1 levels determined by indirect ELISA at 35 and 42 d. (**E**) Lymphocyte proliferation assay results at 35 and 42 d. (**F**) Serum IL-4 levels determined by indirect ELISA at 35 and 42 d. (**G**) Serum IFN-γ levels determined by indirect ELISA at 35 and 42 d. All data are presented as means ± standard deviation; unpaired *t* test with Holm–Sidak adjustment was used for significance analysis, * *p* < 0.05, ** *p* < 0.01, **** *p* < 0.0001, NS, non-significant.

**Figure 7:**
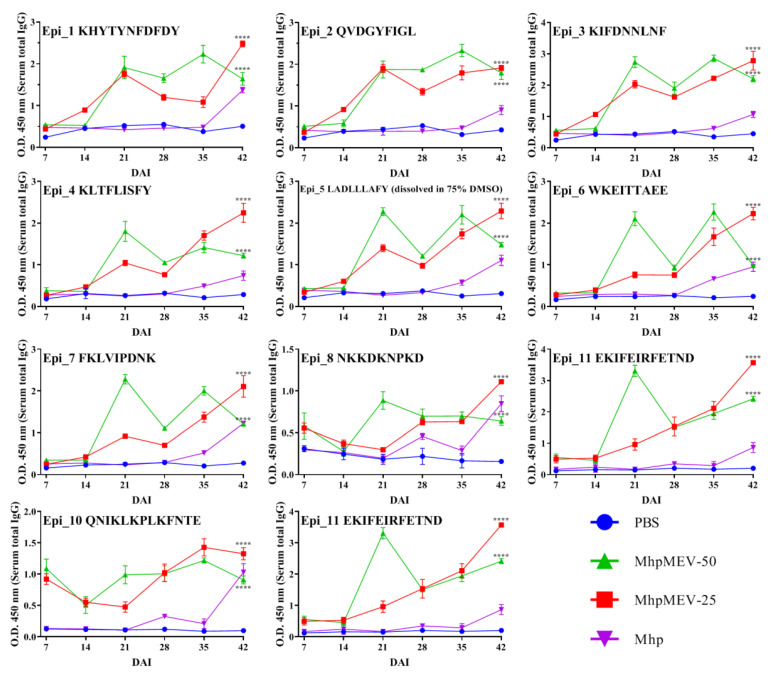
The ELISA results against 11 synthetic epitopes in mouse immune experiment, and the epitope sequences are shown in the top left corner of each figure. Serum antibodies from the MhpMEV-25 group showed higher levels when compared to the MhpMEV-50 group against the 11 synthetic epitopes (*p* < 0.0001), except for QVDGYFIGL at 42 d (ns). The serum antibodies from the Mhp group could also detect all of the synthetic epitopes when compared to the PBS group at 42 d (*p* < 0.0001). The symbol **** indicates a statistical difference between the MhpMEV-25 or MhpMEV-50 group and the PBS group in the figures. All data are presented as means ± standard deviation; unpaired *t* test with Holm–Sidak adjustment was used for significance analysis, **** *p* < 0.0001.

Mouse immunization was performed to evaluate the immunogenicity of MhpMEV, with Freund’s adjuvant used as the immunostimulant. The vaccination schedule of the mouse immune experiment is presented in Figure 6A. The ELISA results of MhpMEV, rMhp597, and the 11 synthetic epitopes are shown in Figure 6B,C and Figure 7, respectively. The levels of serum IgG1 are shown in Figure 6D. There were no significant differences in the ELISA results of MhpMEV (35 d, *p* = 0.2240; 42 d, *p* = 0.0015) and IgG1 levels (35 d, *p* = 0.6283; 42 d, *p* = 0.0062) between MhpMEV-25 and MhpMEV-50 groups. However, at 42 d, serum antibodies from the MhpMEV-25 group showed higher levels against rMhp597 and the 11 synthetic epitopes, except for QVDGYFIGL (*p* < 0.0001), when compared with the MhpMEV-50 group. Moreover, the serum antibodies from the Mhp group could detect all of the synthetic epitopes compared to the PBS group at 42 d (*p* < 0.0001). The results of the lymphocyte proliferation assay are shown in Figure 6E, and the levels of serum IL-4 and IFN-γ are presented in Figure 6F,G. All experimental groups produced higher splenic lymphocyte proliferation and cytokine levels when compared with the PBS group (35 d, *p* < 0.0001; 42 d, *p* < 0.0001). Between the MhpMEV-25 and MhpMEV-50 groups, IFN-γ levels (35 d, *p* = 0.3136; 42 d, *p* = 0.0155) and splenic lymphocyte proliferation showed no significant differences (35 d, *p* = 0.0526; 42 d, *p* = 0.0084), but MhpMEV-50 induced higher levels of IL-4 at 35 and 42 d (*p* < 0.0001, *p* < 0.0001, respectively). 

### 2.10. MhpMEV Induced Humoral Immune Responses and Antiserum Partially Suppressed rMhp597 Nuclease Activity in Piglet Immunization 

**Figure 8:**
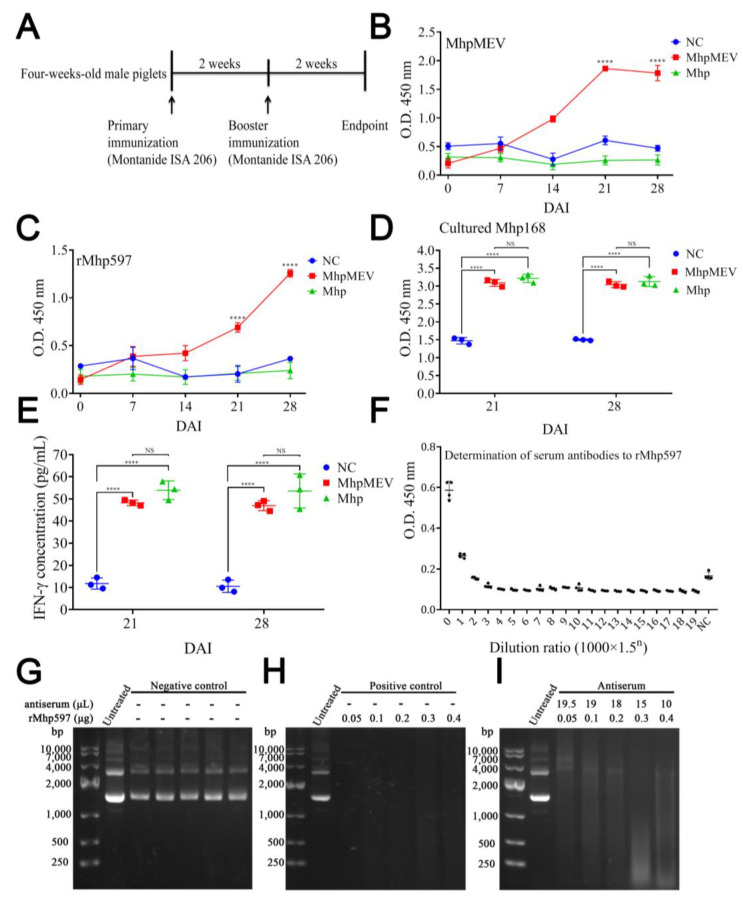
Immunoassay results of piglet immune experiment. (**A**) The vaccination schedule of the piglet immune experiment. (**B**) Serum ELISA results against MhpMEV. (**C**) Serum ELISA results against rMhp597. The symbol **** indicates a statistical difference between the MhpMEV group and the NC group at 21 or 28 d. (**D**) Serum ELISA results against cultured Mhp168 strain at 21 and 28 d. (**E**) Serum IFN-γ levels determined by indirect ELISA at 21 and 28 d. (**F**) Determination of Mhp597 antibody titer in serum at 28 d. Electrophoresis results of different groups of antiserum on the inhibition of rMhp597 nuclease activity are presented in (**G**–**I**), respectively. The concentrations of rMhp597 in reaction are indicated, while the concentrations of antiserum were the opposite. All data are presented as means ± standard deviation; unpaired *t* test with Holm–Sidak adjustment was used for significance analysis, **** *p* < 0.0001, NS, non-significant.

The vaccination schedule of the piglet immune experiment is presented in Figure 8A. The ELISA results of piglet serum antibodies against MhpMEV, rMhp597, and cultured Mhp strain 168 are shown in Figure 8B–D; the serum antibody levels were significantly higher compared to the NC and Mhp groups at 21 and 28 d (*p* < 0.0001). The results of the IFN-γ assay are presented in Figure 8E. The IFN-γ levels in the MhpMEV and Mhp groups were significantly higher than those in the NC group at 21 and 28 d (*p* < 0.0001), but these two groups did not show significant differences. We also determined the serum antibody titer against rMhp597 at 28 d, and the titer was between 1000 and 1500; the result is presented in Figure 8F. To further explore the effectiveness of antibodies induced by MhpMEV, we set up three groups to investigate whether the antiserum could inhibit the nuclease activity of rMhp597, and the results for the different groups are shown in Figure 8G–I. Even the smallest amount of rMhp597 could completely degrade the substrate, but the degradation efficiency could be partially suppressed by adding antiserum.

## 3. Discussion

In the co-evolution of pathogens and host immune systems, pathogens with immune evasion mechanisms have been reported, including influenza A virus (IAV) [23], SARS-CoV-2 [24], *Streptococcus pneumoniae* [25], and herpes simplex virus 1 [26]. We proposed earlier that genomic variability and post-translational protein modification enhance the immune evasion of Mhp [5], which limits the general applicability of commercial vaccines. On the other hand, reverse vaccinology has been developed for a rapid and effective vaccine development pipeline in recent studies, and a variety of candidate vaccines against viruses, parasites, and bacteria have been reported. In the reverse vaccinology pipeline, pan-genome analysis can find ubiquitous antigens within different strains. The selected immunodominant antigens only account for 1% of all core proteins in this study. In another study, this proportion was also very low (2212 core proteins and 4 immunodominant antigens in *Klebsiella pneumoniae*), which indicates that the pipeline for candidate antigen selection is strict [27]. These antigens can be used for epitope selection and can also serve as candidate immunogens for genetically engineered vaccines.

The steps for epitope selection in this study mainly included selecting B-cell, MHC-II binding, and MHC-I binding epitopes. These processes were rapid, and, to improve the accuracy, more than one prediction or identification method was used. The final vaccine was a combination of conserved epitopes rather than subunit proteins, and the results of multiple sequence alignments of epitopes and their homologous sequences in different Mhp strains are shown in Supplementary File S1. Although some epitopes had amino acid mutations, the frequency of these mutations was relatively low. These properties may enable a multi-epitope vaccine to induce universal antibodies against the selected antigens and overcome the immune evasion of Mhp. 

The AAYAAY linker was used to integrate the MHC-I/II binding epitopes to produce MhpMEV; other linkers, including AAY [28], GPGPG [27], EAAAK [29], and GGGGS [30], have been used in previous studies, while the AAYAAY linker was reported to induce a higher level of specific antibodies [30]. For the structural analysis, MhpMEVC6His can be regarded as an artificial protein, because no homologous protein was found in the NCBI nr database. By the analysis of circular dichroism, MhpMEVC6His was found to have more random coils and antiparallels, but, in in silico structural analysis, alpha helix occupied most of the secondary structure. Moreover, a Ramachandran plot analysis showed that Robetta could provide a more suitable model than I-TASSER and AlphaFold2, but all of the predicted three-dimensional models showed significant differences when compared with the results of the circular dichroism analysis. These results indicate that the structure prediction methods for artificial proteins still need further study. 

Freund’s adjuvant is an oil adjuvant widely used in animal vaccines, but its ability to stimulate the production of antibodies against recombinant protein is not good, and it often leads to excessive inflammatory responses [31]. Because of this, we used MontanideTM ISA 206 VG adjuvant in the piglet immunization experiments. We found that the emulsion formed from MontanideTM ISA 206 VG was more stable compared to incomplete Freund’s adjuvant at 4 and 20 °C [32]. In the mouse immune experiment, ELISA results showed that the serum antibodies induced by MhpMEV could detect MhpMEV, rMhp597, and the 11 synthetic epitopes. Moreover, the spleen lymphocyte proliferation and cytokine secretion levels (IFN-γ and IL-4) of the MhpMEV-25 and MhpMEV-50 groups were higher than those of the PBS group (*p* < 0.0001). IFN-γ production enhanced the activity of Th1 cells and promoted cellular immune responses, while the Th2-type immune response was mainly regulated by the secretion of IL-4 [33,34]. Cytokine detection results indicated that MhpMEV could induce a mixed Th1/Th2 immune response, further demonstrating that MhpMEV was able to induce humoral and cellular immune responses in mice. On the other hand, epitope 6 (WKEITTAEE) was predicted to have the lowest immunogenicity, as shown in Figure 3A, but in immunized mice and piglets, this epitope was able to induce antibodies against the synthetic peptide and rMhp597. This might be because the use of the adjuvant increased the immunogenicity of this epitope, while the VaxiJen prediction method only considered the immune response of the peptide itself. It should be emphasized that the piglet antiserum partially suppressed the nuclease activity of the immunodominant antigen rMhp597, even if the antibodies were induced by one epitope and the antibody titer was only between 1000 and 1500. 

Current vaccines lack protective efficacy, and although they can reduce clinical symptoms, they cannot prevent infection by Mhp or its spreading. The strain variability may partly explain this, but local immunodeficiency due to parenteral vaccine injection also contributes [35,36]. In this study, immune experiments with both mice and piglets provided experimental data for the effectiveness of MhpMEV, revealing that it was able to induce cellular and humoral immune responses. The key limitation of this research is that the protective effect of MhpMEV was not evaluated under the bacterial burden or with co-infection by Mhp and other pathogens; in addition, the duration of the vaccine’s protective efficacy still requires further investigation.

## 4. Materials and Methods

### 4.1. Pan-Genome Analysis and Prioritization of Immunodominant Virulence Factors

Genome GBFF files of 21 Mhp strains were retrieved from the National Center for Biotechnology Information (NCBI) database. The files were converted to GFF3 files with BioPerl bp_genbank2gff3.pl script and used as input files for pan-genome analysis. The Roary installation and dependency checks were done on a Bio-Linux 8 system, and the pan-genome analysis was performed [37,38]. Afterward, the core proteins from Mhp strain 232 were selected for further analysis. 

To minimize the chance of autoimmunity or an inadequate immune response, the core proteins were aligned to the *Sus scrofa* (taxid: 9823) proteome using the online BLASTp program. The homologous proteins were identified as percent identity > 35, bit score > 100, and e-value < 1.0 ×10^−5^, and homologous proteins were removed from the core proteins [27]. For subcellular localization, candidate proteins were submitted to Psortb version 3.0 [39] to select outer membrane proteins, and the unknown proteins were further uploaded to CELLO2GO [40] to identify the outer membrane proteins, and the outer membrane proteins predicted by the two services were combined. Virulence factors within the outer membrane proteins were identified by alignment with the Virulence Factor Database (VFDB) [41] using the local BLASTp program. The basic physical properties of the screened virulence factors were calculated using the ProtParam tool in the ExPASy portal [42]. Trans-membrane helices and signal peptides were predicted by TMHMM server V2.0 [43] and SignalIP-5.0 [44], respectively. The immunogenicity of these proteins was calculated by the Kolaskar and Tongaonkar method [45], ANTIGENpro [46], and VaxiJen V2.0 [47] with default parameters. BepiPred 2.0 was used to predict the immunogenicity of each amino acid in these proteins [48]. 

### 4.2. Prediction of B-Cell Epitopes

ABCPred [49] was used to identify the potential B-cell epitopes within the immunodominant antigens, and the threshold was set to 0.51. To improve accuracy, Bcepred [50] was used to screen the linear B-cell epitopes, and this method was based on peptide physicochemical properties, including hydrophilicity, flexibility, exposed surface, and polarity. The peaks of the amino acid residue segments above the threshold (1.9, 2, 2.3, and 1.8 for each property, respectively) were considered as potential linear B-cell epitopes. BLASTp was used to find the co-identified epitopes by the two services, and duplicate epitopes were removed.

### 4.3. Prioritization of B-Cell-Derived MHC-II Binding Epitopes

The methods for selecting MHC-II binding epitopes were mainly based on a published study, with appropriate modifications [27]. The B-cell epitopes were submitted to EpiTOP 3.0 [51], a proteochemometrics tool for MHC-II binding prediction. The locus was selected as “HLA-DRB1”, and protein segments that could bind three or more alleles and with pCI50 above 6.3 were selected as potential MHC-II binding epitopes. The predicted MHC-II binding epitopes were further submitted to VaxiJen V2.0 [47] to evaluate their antigenicity; the model was set to bacteria and the threshold was 0.5. Then, the shortlisted epitopes were uploaded to MHCPred version 2.0 [52] to evaluate their binding affinity to predominant HLA-II allele DRB1*0101, and those with IC50 < 500 nM were shortlisted [53]. VirulentPred predicted the cytotoxicity of these epitopes [54]; then, the epitopes with virulent predictions were submitted to IFNepitope [55] to determine whether they could induce IFN-γ production, and those with positive results were selected as MHC-II binding epitopes.

### 4.4. Prioritization of MHC-I Binding Epitopes

MHC-I binding prediction v2.23 was used to find potential MHC-I binding epitopes within the immunodominant antigens using the ANN 4.0 method [56]. Porcine MHC alleles SLA-1*0401, SLA-1*0701, SLA-2*0401, and SLA-3*0401 were selected as receptors. The epitope length was set to 9 amino acids, and epitopes with calculated IC50 < 500 nM were selected as potential MHC-I binding epitopes. The immunogenicity of these epitopes was calculated by the MHC I immunogenicity score, a tool validated for peptides of 9 amino acids in length [57], and epitopes with a predicted score > 0 were shortlisted. To further increase the credibility, the epitopes were evaluated for their binding affinity to predominant HLA-I allele HLA-A*0201 by MHCPred version 2.0 [52], and those with a calculated IC50 < 500 nM were selected [53]. Finally, MHC-I binding epitopes were identified based on their antigenic propensity predicted by VaxiJen V2.0 [47] with default parameters. 

### 4.5. Multi-Epitope Vaccine Design and Protein Expression

The MHC-I/II binding epitopes containing overlapping sequences were merged, and they were combined by the AAYAAY linker to produce an integrated recombinant protein, MhpMEV. The MHC-I binding epitopes were placed at the N-terminal and the MHC-II binding epitopes were placed at the C-terminal. For protein expression, codon optimization for MhpMEV was performed by GenScript, and the DNA sequence was synthesized. Then, it was inserted into the *Bam*H I and *Eco*R I restriction sites of pET22b, the 6×His Tag was retained, and GenScript provided the final recombinant plasmid. *E. coli* BL21(DE3) competent cells were prepared by 0.1 mol/L CaCl_2_ treatment on ice, and the recombinant plasmid was transformed by heating at 42 °C for 90 s. The cells were plated on solid LB medium containing 20 μg/mL ampicillin for selection. For protein expression, recombinant *E. coli* BL21(DE3) cells were cultured in 50 mL LB medium containing 20 μg/mL ampicillin at 37 °C, and when the OD600 value reached 0.6, four culture conditions were used for MhpMEV expression: ① 0.1 mM IPTG at 18 °C for 24 h, ② 0.2 mM IPTG at 18 °C for 24 h, ③ 0.1 mM IPTG at 25 °C for 18 h, and ④ 0.2 mM IPTG at 25 °C for 18 h. After incubation, the pellets were collected and lysed by sonication on ice. The supernatant and sedimentation were separated and loaded for 12% SDS-PAGE. The immunogenicity of each amino acid in MhpMEV was calculated by BepiPred 2.0 [48]. 

### 4.6. Western Blotting

The expression of MhpMEV was identified by Western blotting using a previously described method [58]. After protein expression, the sedimentation was resuspended in PBS buffer containing different concentrations of urea (1–8 mol/L) and loaded for 12% SDS-PAGE. Mouse monoclonal anti-His antibody (1:1000, Beyotime, Shanghai, China) was used as the primary antibody, and the secondary antibody was HRP-labeled goat anti-mouse IgG (H+L) (1:2000, Beyotime, Shanghai, China). The assays were developed using an enhanced chemiluminescence (ECL) method (Advansta, San Jose, CA, USA), following the manufacturer’s instructions.

### 4.7. Denaturation and Refolding of MhpMEV Inclusion Bodies

Recombinant *E. coli* BL21(DE3) was cultured in 2 L LB medium containing 20 μg/mL ampicillin. When the OD600 value reached 0.6, MhpMEV was induced by the addition of 0.2 mM IPTG and incubated at 18 °C for 24 h. The pellet was collected and lysed by sonication on ice, and the inclusion bodies were separated by centrifugation at 12,000 rpm for 30 min. Then, WB-I (50 mM Tris-HCl, 100 mM NaCl, 10 mM EDTA, 1% Triton X-100, 100 mM β-mercaptoethanol, pH 8.0), WB-II (WB-I + 0.3 mol/L urea), and WB-III (WB-I + 0.6 mol/L urea) washing buffers were used to wash the inclusion bodies. The sediment was collected following centrifugation at 12,000 rpm for 10 min after each washing step. After thorough washing, MhpMEV was dissolved in 8 mol/L urea and centrifuged to remove insoluble matter. For protein refolding, MhpMEV was continuously dialyzed in a 3500 D dialysis membrane in the following dialysates: RB-I (RB-IV + 6 mol/L urea), RB-II (RB-IV + 4 mol/L urea), RB-III (RB-IV + 2 mol/L urea), and RB-IV (50 mM Tris-HCl, 5% glycerol, pH 8.0). The time for each dialysis step was 3 h at 4 °C, and the total dialysis time was more than 12 h. After protein refolding, MhpMEV was collected following centrifugation at 12,000 rpm for 30 min. The purity and concentration were determined by BCA and 12% SDS-PAGE. MhpMEV was concentrated to 1 mg/mL by ultrafiltration and stored at −80 °C until use.

### 4.8. Structure Analysis

To improve the purity of MhpMEV, it was redesigned as MhpMEVC6His and expressed in *E. coli*. After protein refolding, MhpMEVC6His was purified by nickel ion affinity chromatography and dialyzed in 50 mM Tris-HCl and 5% glycerol (pH 8.0) to remove imidazole. MhpMEVC6His was sent to Sangon Biotech for the circular dichroism assay. At the same time, there were no homologous sequence hits for MhpMEVC6His by the BLASTp program within the NCBI NR database, so the secondary structure was predicted by RaptorX Property [59], since it could predict the structural properties without using any templates. For three-dimensional structure prediction, Robetta, I-TASSER, and AlphaFold [17] were used [60,61]. For the validation of models [62], ProSA-web [63] and PDBsum [64] were used, and the molecular models were visualized by PyMOL version 1.8.2.0.

### 4.9. Molecular Docking

ZDOCK [65] is an interactive docking prediction service for protein–protein complexes and symmetric multimers. In this study, ZDOCK was used to find the interactions between MhpMEVC6His and swine MHC class I antigens (3QQ4, 5YLX, 6A6H, and 7EMA) or Toll-like receptor 3 (2A0Z), which were downloaded from the Protein Data Bank (PDB). After calculation, the top 10 best binding models for each receptor were provided based on ZDOCK scores. The best binding model of MhpMEVC6His and Toll-like receptor 3 was visualized by PyMOL version 1.8.2.0.

### 4.10. Ethical Statement on Animal Immunization

Although the molecular docking results indicated that MhpMEVC6His could bind to immune-associated receptors, we performed animal immunization to validate our findings and studied the immune response. The design of the animal immunization was based on our previously published papers [31,66,67]. All animals (cleanliness of mice: SPF; cleanliness of piglets: free from Mhp and porcine circovirus type 2 infection) were kept in individually ventilated cages. The Experimental Animal Welfare Ethics Committee of Zhejiang Sci-Tech University approved the experimental methods (mouse immune experiment acceptance number: 20201010-08; piglet immune experiment acceptance number: 20210620-15), and care was given according to the 3R principle during the entire investigation [68].

### 4.11. Immunization of Mice

Thirty-two 6-to-8-week-old female BALB/c mice (SPF-grade; purchased from Shanghai SLAC Laboratory Animal Co., Ltd., Shanghai, China) were randomly divided into four groups, with eight animals per group. Grouping and vaccination strategies were as follows: ① PBS group (negative control, injected with sterile PBS), ② MhpMEV-25 group (each mouse immunized with 25 μg MhpMEV per immunization), ③ MhpMEV-50 group (each mouse immunized with 50 μg MhpMEV per immunization), and ④ Mhp group (commercial swine Mycoplasma pneumonia vaccine, live 168 strain; JOFUNHWA Biotechnology Co., Ltd., Nanjing, China). For the MhpMEV-25 and MhpMEV-50 groups, the MhpMEV stock solution was diluted and emulsified with isometric Freund’s adjuvant (Sigma, St. Louis, MO, USA). The emulsification was prepared according to a previously published method and used on the day of the experiment [69]. For the Mhp group, the commercial vaccine was resuspended in the provided special diluent and stored at −20 °C until use. Multi-point subcutaneous injection was performed in the back with 200 μL of immunogen in the 4 groups on days 0, 14, and 28. In the MhpMEV-25 and MhpMEV-50 groups, Freund’s complete adjuvant was used for the first immunization, and Freund’s incomplete adjuvant was used for the two booster immunizations. Blood collection and serum separation were performed every week, and all serum samples were stored at −80 °C until use. 

### 4.12. Immunization of Piglets 

Fifteen 4-week-old male piglets weighing 7–8 kg were randomly divided into three groups with five piglets per group: negative control group (NC, inoculated with 2 mL sterile normal saline on days 0 and 14 through intramuscular neck injection for each immunization), MhpMEV group (immunized twice on days 0 and 14; 1 mg/mL MhpMEV and isometric MontanideTM ISA 206 VG (Seppic, Courbevoie, France) were mixed and emulsified, and 2 mL emulsion was administered through intramuscular injection), and Mhp group (inoculated with 1 mL commercial vaccine (swine Mycoplasma pneumonia vaccine, live 168 strain; JOFUNHWA Biotechnology Co., Ltd.) through intramuscular neck injection on days 0 and 14). MontanideTM ISA 206 VG adjuvant consisted of water, oil, and surfactants, with high stability and only mild side effects [70]. It is the most widely used adjuvant for animal vaccines [71,72]. The animal experiment lasted for four weeks, and serum samples were collected every week and stored at −80 °C until use. 

### 4.13. Enzyme-Linked Immunosorbent Assay (ELISA)

To detect the serum antibodies, 11 epitopes were synthesized by Sangon Biotech Co., Ltd. (Shanghai, China), with 75% purity. Recombinant proteins MhpMEV and rMhp597 and culture Mhp strain 168 [67] were also used as antigens. The ELISA assay was performed according to a previously published method [58,73]. Briefly, the plate was coated with 100 μL antigen (1 μg/mL) at 4 °C overnight. The next day, the plate was blocked by 5% BSA in PBS at 37 °C for 2 h. The collected serum was diluted at 1:200 with antibody dilution (2% skim milk powder and 3% bovine serum albumin in PBS), and the plate was incubated with 100 μL diluted serum for 2 h at 37 °C. After washing the plate, the secondary antibodies were diluted by the same antibody dilution, and 100 μL of secondary antibodies was added and incubated at 37 °C for 1 h. The secondary antibodies were HRP-labeled goat anti-mouse IgG (H+L) (1:2000, Beyotime, Shanghai, China) and HRP-labeled goat anti-pig IgG (1:2000, Solarbio, Beijing, China) for serum samples collected from mice and piglets, respectively. TMB single-component (100 μL) substrate solution was added, and the OD450 was read with a multifunction microplate reader (Bio-Rad, Hercules, CA, USA). The levels of mouse serum IgG1 and cytokines (IL-4 and IFN-γ) were detected by Mouse IgG1/IL-4/IFN-γ ELISA Kits (Solarbio, China) at days 35 and 42, respectively. The levels of piglet serum IFN-γ were determined by the Pig IFN-γ ELISA Kit (Jiangsu MEIMIAN Industry Co., Ltd., Yancheng, China), and the serum antibody titer against rMhp597 was determined at 28 d for the MhpMEV group. 

### 4.14. Lymphocyte Proliferation Assay

The lymphocyte proliferation assay was performed on days 35 and 42 for the mouse immune experiment, as described previously [74]. The splenic lymphocytes were isolated by Mouse Lymphocyte Separation Solution (DAKEWEI, Beijing, China) according to the manufacturer’s instructions and seeded into 96-well plates at 5 × 105 cells/well. Briefly, 2 μg of filter-sterilized MhpMEV was added to 100 μL of RPMI 1640 medium (Gibco, Waltham, MA, USA) as a stimulator, 100 μL of RPMI 1640 medium containing 2 μg of concanavalin A (Sigma, St. Louis, MO, USA) was added as a positive control, and 100 μL of RPMI 1640 medium was added as a negative control. The cells were treated with different stimulators for 36 h. Each splenic lymphocyte sample was plated in triplicate, and the proliferative activity was measured by a standard MTT assay (5 mg/mL; Solarbio, China). The OD490 was read with a multifunction microplate reader (Bio-Rad, USA), and the stimulation index (SI) was calculated as follows: SI = (mean OD of MhpMEV treated cells − mean OD of negative control)/(mean OD of positive control − mean OD of negative control). 

### 4.15. Suppression Assay of Piglet Antiserum to rMhp597 Nuclease Activity

The piglet serum collected on day 28 was used to determine the antibody titer against rMhp597 according to a previously published method [66]. To further explore the inhibitory effect of serum antibodies induced by a single epitope from rMhp597, we set up three groups to investigate: positive control, negative control, and antiserum groups. Each group had the same concentration of pUC57 plasmid (extracted directly from *E. coli* host as the substrate), Ca^2+^, and Mg^2+^. The rMhp597 (0.1 mg/mL) and serum samples collected on day 28 of piglet immunization were used. The reaction systems are presented in Supplementary File S4 Table S3. The tubes were incubated at 37 °C for 2 min and stopped by heating in a 95℃ water bath; then, 1% agarose gel nucleic acid electrophoresis was performed immediately. 

## 5. Conclusions

In the current study, we developed a multi-epitope vaccine for MPS, named MhpMEV, based on the reverse vaccinology pipeline. The secondary structure was determined by circular dichroism, and the three-dimensional structure was predicted by Robetta, I-TASSER, and AlphaFold. The Robetta service gave a more suitable protein model, but it still showed significant differences when compared with the circular dichroism analysis results. Through molecular docking, MhpMEVC6His was able to induce immune responses. In immunized mice and piglets, MhpMEV induced humoral and cellular immune responses. Briefly, this study provides wet-lab immunoassay results of a multi-epitope vaccine derived from the reverse vaccinology pipeline and provides a conceptual multi-epitope vaccine for MPS. 

## Data Availability

The original data presented in the study are included in the article and Supplementary Files. Further inquiries can be directed to the corresponding author.

## Supplementary Materials

The following supporting information can be downloaded at: https://www.mdpi.com/article/10.3390/ijms23147899/s1. References [6,7,8,19,20,21,31,75,76,77,78,79,80,81,82,83,84,85,86,87,88] are cited in the supplementary materials.
