# Supplementary material for: Development of a Multi-Epitope Vaccine for Mycoplasma hyopneumoniae and Evaluation of Its Immune Responses in Mice and Piglets
Source: Int J Mol Sci. 2022 Jul 18;23(14):7899. doi: 10.3390/ijms23147899 (PMC9318870; doi:10.3390/ijms23147899)
Supplement: Supplementary file 1 [file ijms-23-07899-s001.zip › ijms-1768532-supplementary/Supplementary File 1.pdf]

Supplementary File S1

The virulence factors used in sequence alignment mainly included adhesins, chaperone proteins, membrane proteins or cytosolic proteins, the proteins were collected from the published papers and listed in **Table S1**. These sequences were aligned to local Mhp proteome database (the proteome data set of twenty-one Mhp strains as used in the pan-genome analysis) with BLASTp program. The homologous sequences were identified as percentage identity > 35%, bit score > 100 and e-value < $1.0 \times e^{-5}$. It was worth noting that the number of homologous proteins was usually an integer multiple of 21, and indicated that some virulence factors had multiple copies in the genome. After obtaining the homologous proteins, the multiple sequence alignment and phylogenetic tree construction were performed by ClustalW (Larkin et al., 2007). The phylogenetic trees were visualized by iTOL v6 (https://itol.embl.de/) and presented in **Figure S1** (Letunic and Bork, 2021). What's more, sequence alignment results of the repeat segments from P146 and P97 were presented in **Figure S2A** and **Figure S2B**, respectively. P146 and P97 were usually used as molecules for Mhp genome classification (Vranckx et al., 2011; Felde et al., 2018; Betlach et al., 2019) and vaccines research (Conceicao et al., 2006; Bogema et al., 2012; de Oliveira et al., 2017), while protein variety in different Mhp strains may decrease the universal applicability of the vaccines that developed based on these antigens.

Num	Protein	Representative accession number in NCBI	Category	Total number of homologous proteins in 21 Mhp strains	Reference
1	P97	AAB47Q806.1	Adhesion factor	43	(Minion et al., 2000)
2	P102	AAC32527.1	Adhesion factor	64	(Adams et al., 2005)
3	P116	AAV27417.1	Adhesion factor	21	(Seymour et al., 2010)
4	P216	AAQ11369.1	Adhesion factor	27	(Tacchi et al., 2014)
5	P146	AAF91425.1	Adhesion factor	28	(Bogema et al., 2012)
6	P159	AAV27918.1	Adhesion factor	22	(Raymond et al., 2013)
7	Mhp107	WP_179221081.1	Adhesion factor	22	(Seymour et al., 2011)
8	Mhp384	AAV27855.1	Adhesion factor	23	(Deutscher et al., 2012)
9	Mhp385	AAV27856.1	Adhesion factor	19	(Deutscher et al., 2012)
10	P46	ADQ90718.1	Membrane protein	21	(Galli et al., 2012; Guasch et al., 2020)
11	P65	ABE73147.1	Membrane lipoprotein	23	(Schmidt et al., 2004)
12	Mhp366	AAV27366.1	Membrane protein	66	(Meens et al., 2010)
13	DnaK	WP_160606515.1	Chaperone protein DnaK	23	(Galli et al., 2012; Tao et al., 2020)
14	P36	AXP07918.1	Cytosolic protein	22	(Caron et al., 2000)

Table S1. The representative virulence factors used in the sequence alignment and phylogenetic treeconstruction

1 - P97

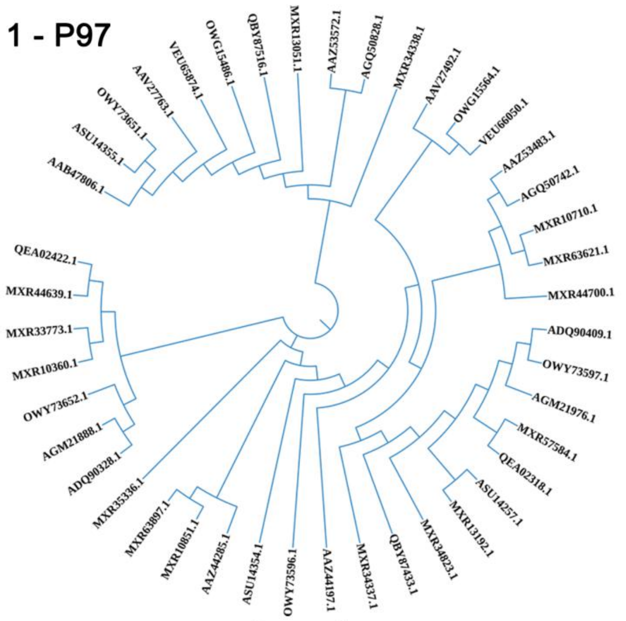

2 - P102

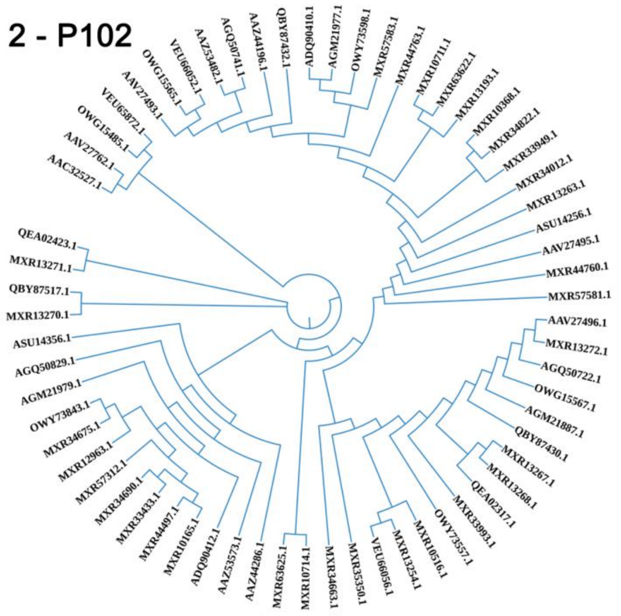

3 - P116

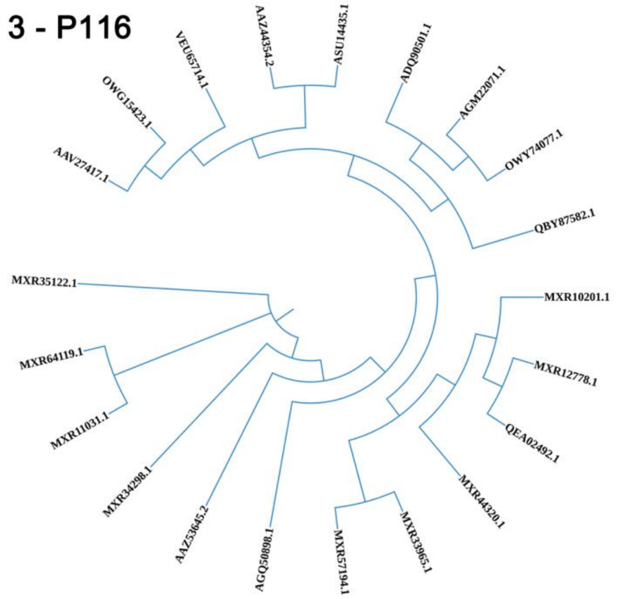

4 - P216

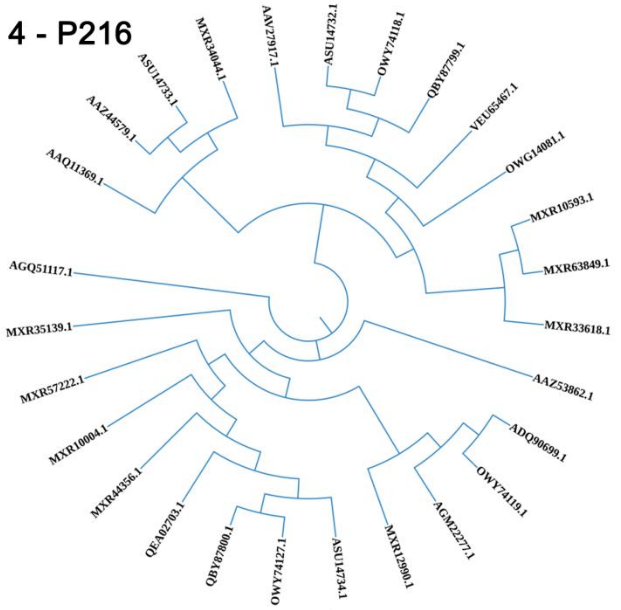

5 - P146

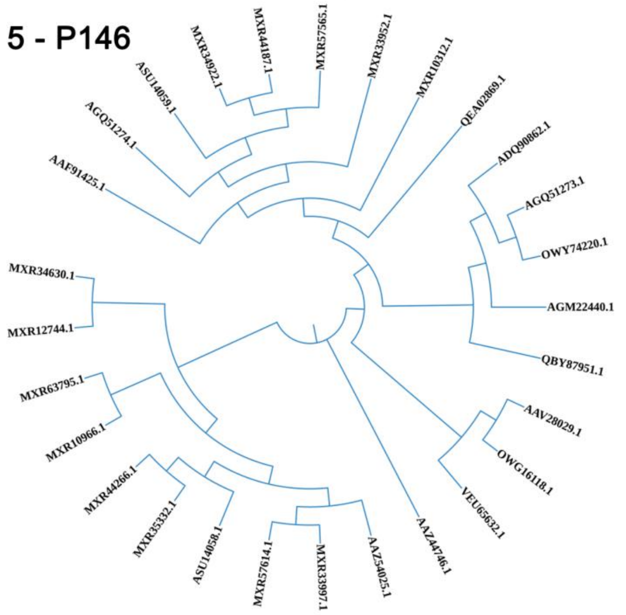

6 - P159

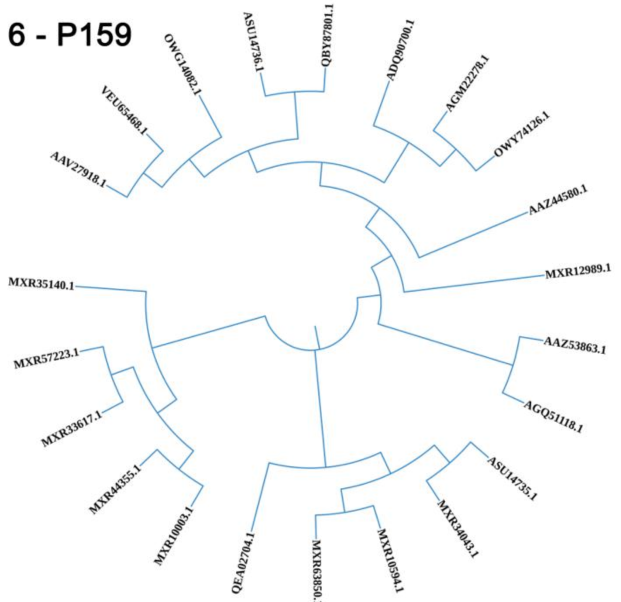

7 - Mhp107

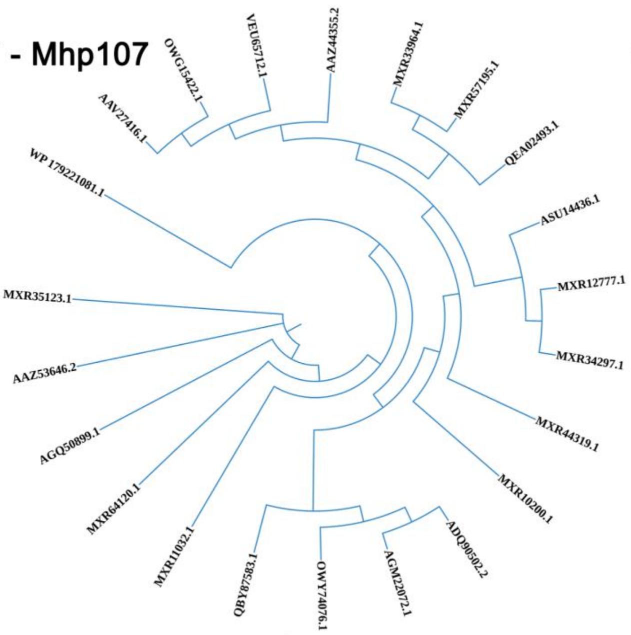

8 - Mhp384

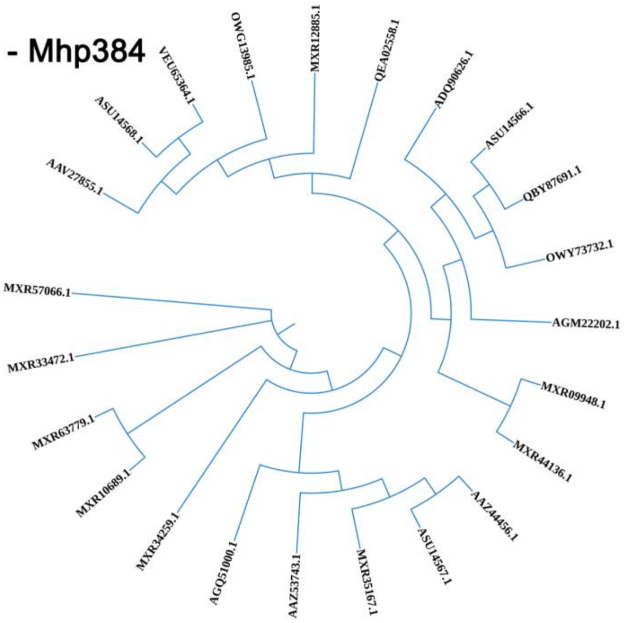

9 - Mhp385

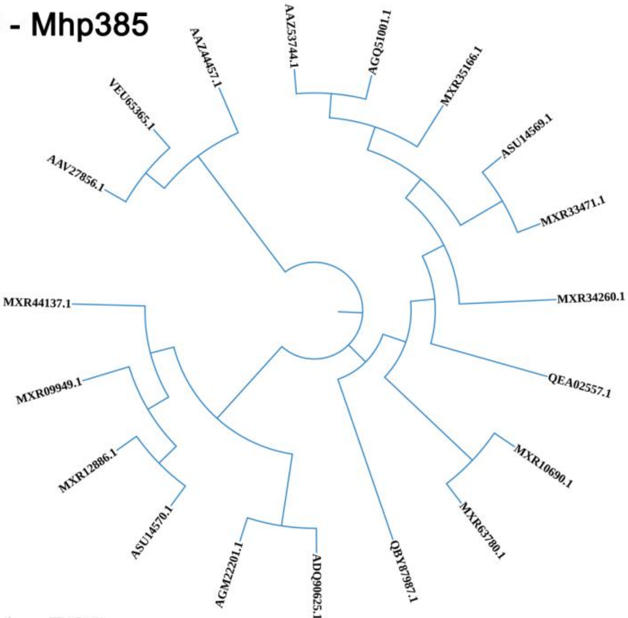

10 - P46

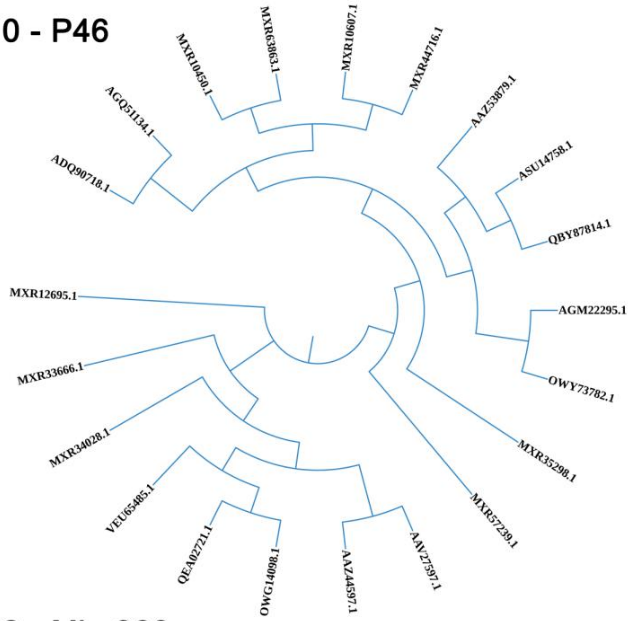

11 - P65

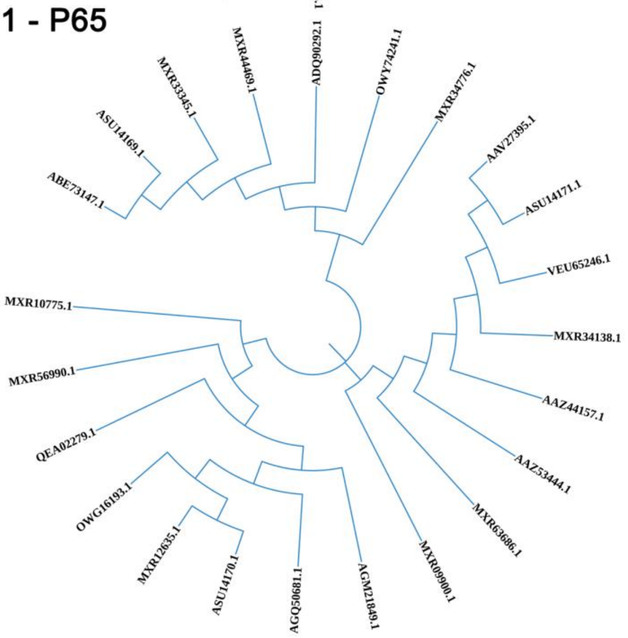

12 - Mhp366

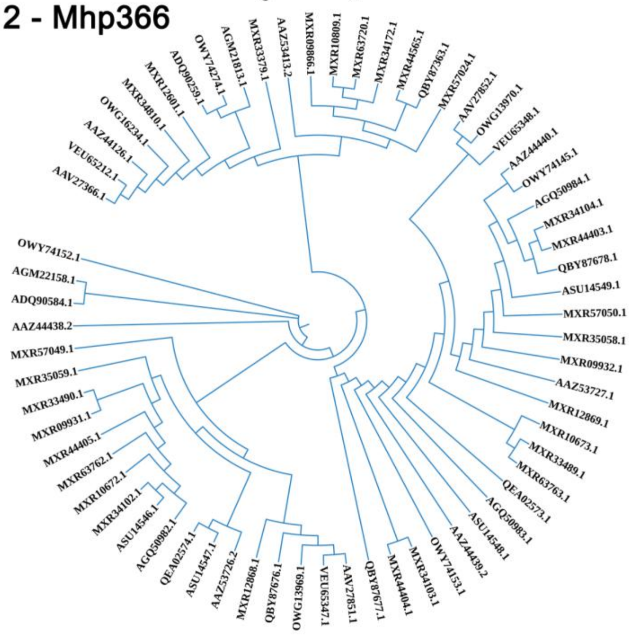

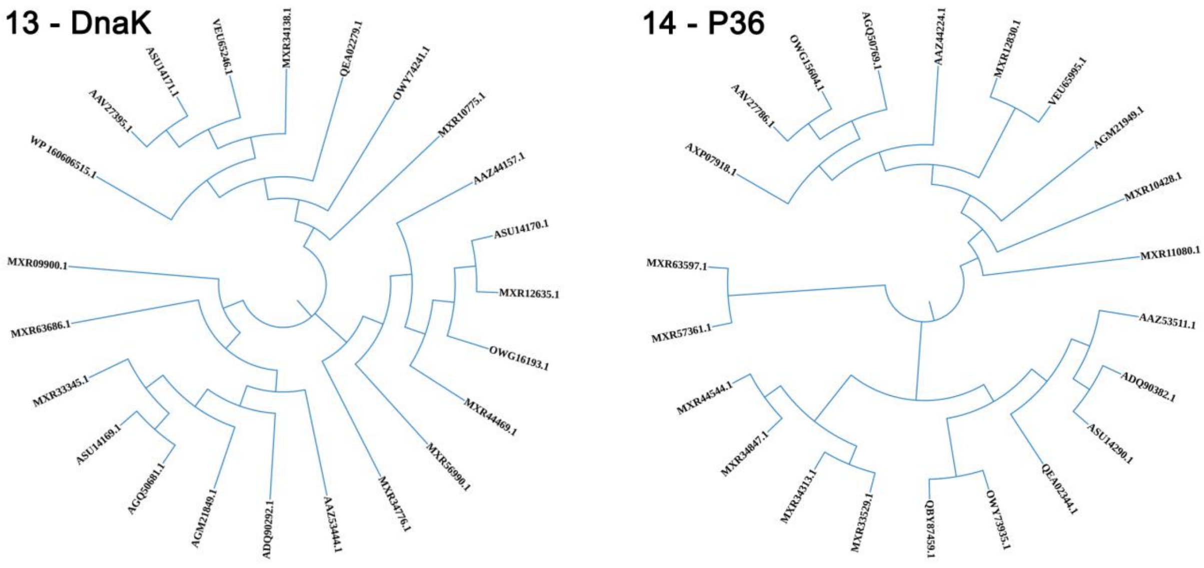

Figure S1 Phylogenetic tree construction of the 14 virulence factors with their homologous proteins. The homologous proteins were obtained by BLASTp program within the twenty-one Mhp strains. The multiple sequence alignment was performed by ClustalW and the phylogenetic trees were visualized by iTOL.

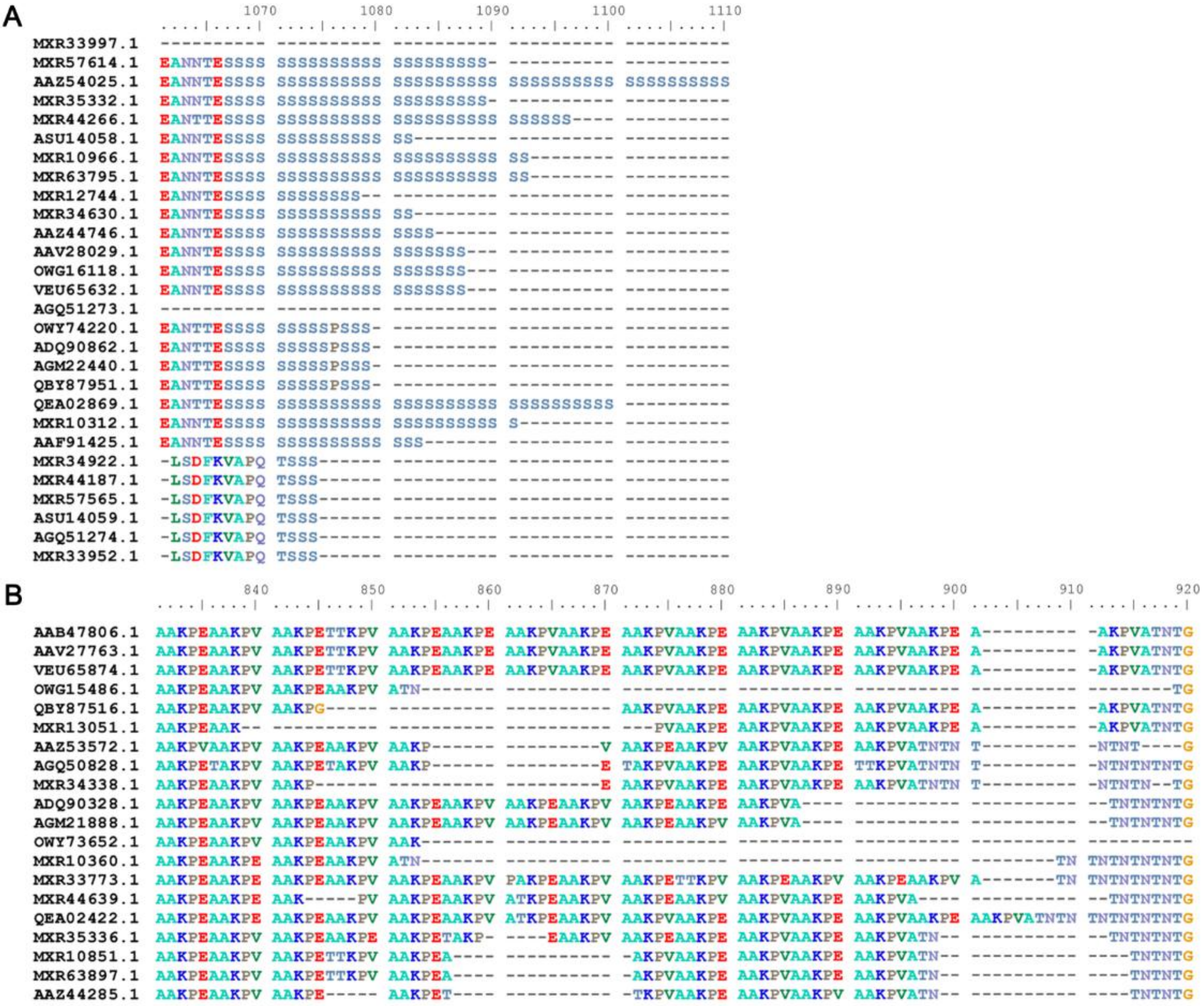

Figure S2 The sequence alignment results of the repeat segments of P146 and P97. **(A)** P146 had a continuous repeating sequence of serine. **(B)** The repetitive segment of P97 was AAKPE/V at its C-terminus.

Reference

Adams, C., Pitzer, J., and Minion, F.C. (2005). *In vivo* expression analysis of the P97 and P102 paralog families of *Mycoplasma hyopneumoniae*. *Infect Immun* 73(11):7784-7787. doi: 10.1128/IAI.73.11.7784-7787.2005.

Betlach, A.M., Maes, D., Garza-Moreno, L., Tamiozzo, P., Sibila, M., Haesebrouck, F., et al. (2019). *Mycoplasma hyopneumoniae* variability: Current trends and proposed terminology for genomic classification. *Transbound Emerg Dis* 66(5):1840-1854. doi: 10.1111/tbed.13233.

Bogema, D.R., Deutscher, A.T., Woolley, L.K., Seymour, L.M., Raymond, B.B., Tacchi, J.L., et al. (2012). Characterization of cleavage events in the multifunctional cilium adhesin Mhp684 (P146) reveals a mechanism by which *Mycoplasma hyopneumoniae* regulates surface topography. *mBio* 3(2):e00282-11. doi: 10.1128/mBio.00282-11.

Caron, J., Sawyer, N., Ben Abdel Moumen, B., Cheikh Saad Bouh, K., and Dea, S. (2000). Species-specific monoclonal antibodies to *Escherichia coli*-expressed p36 cytosolic protein of *Mycoplasma hyopneumoniae*. *Clin Diagn Lab Immunol* 7(4):528-535. doi: 10.1128/cdli.7.4.528-535.2000.

Conceicao, F.R., Moreira, A.N., and Dellagostin, O.A. (2006). A recombinant chimera composed of R1 repeat region of *Mycoplasma hyopneumoniae* P97 adhesin with *Escherichia coli* heat-labile enterotoxin B subunit elicits immune response in mice. *Vaccine* 24(29-30):5734-5743. doi: 10.1016/j.vaccine.2006.04.036.

de Oliveira, N.R., Jorge, S., Gomes, C.K., Rizzi, C., Pacce, V.D., Collares, T.F., et al. (2017). A novel chimeric protein composed of recombinant *Mycoplasma hyopneumoniae* antigens as a vaccine candidate evaluated in mice. *Vet Microbiol* 201:146-153. doi: 10.1016/j.vetmic.2017.01.023.

Deutscher, A.T., Tacchi, J.L., Minion, F.C., Padula, M.P., Crossett, B., Bogema, D.R., et al. (2012). *Mycoplasma hyopneumoniae* Surface proteins Mhp385 and Mhp384 bind host cilia and glycosaminoglycans and are endoproteolytically processed by proteases that recognize different cleavage motifs. *J Proteome*

Res 11(3):1924-1936. doi: 10.1021/pr201115v.

Felde, O., Kreizinger, Z., Sulyok, K.M., Marton, S., Banyai, K., Korbuly, K., et al. (2018). Genotyping *Mycoplasma hyopneumoniae* isolates based on multi-locus sequence typing, multiple-locus variable-number tandem repeat analysis and analysing gene p146. *Vet Microbiol* 222:85-90. doi: 10.1016/j.vetmic.2018.07.004.

Galli, V., Simionatto, S., Marchioro, S.B., Fisch, A., Gomes, C.K., Conceicao, F.R., et al. (2012). Immunisation of mice with *Mycoplasma hyopneumoniae* antigens P37, P42, P46 and P95 delivered as recombinant subunit or DNA vaccines. *Vaccine* 31(1):135-140. doi: 10.1016/j.vaccine.2012.10.088.

Guasch, A., Montané, J., Moros, A., Piñol, J., Sitjà, M., González-González, L., et al. (2020). Structure of P46, an immunodominant surface protein from *Mycoplasma hyopneumoniae*: interaction with a monoclonal antibody. *Acta Crystallogr D Struct Biol* 76(Pt 5):418-427. doi: 10.1107/s2059798320003903.

Larkin, M.A., Blackshields, G., Brown, N.P., Chenna, R., McGettigan, P.A., McWilliam, H., et al. (2007). Clustal W and Clustal X version 2.0. *Bioinformatics* 23(21):2947-2948. doi: 10.1093/bioinformatics/btm404.

Letunic, I., and Bork, P. (2021). Interactive Tree Of Life (iTOL) v5: an online tool for phylogenetic tree display and annotation. *Nucleic Acids Res* 49(W1):W293-W296. doi: 10.1093/nar/gkab301.

Meens, J., Bolotin, V., Frank, R., Bohmer, J., and Gerlach, G.F. (2010). Characterization of a highly immunogenic *Mycoplasma hyopneumoniae* lipoprotein Mhp366 identified by peptide-spot array. *Vet Microbiol* 142(3-4):293-302. doi: 10.1016/j.vetmic.2009.10.007.

Minion, F.C., Adams, C., and Hsu, T. (2000). R1 region of P97 mediates adherence of *Mycoplasma hyopneumoniae* to swine cilia. *Infect Immun* 68(5):3056-3060. doi: 10.1128/IAI.68.5.3056-3060.2000.

Raymond, B.B., Tacchi, J.L., Jarocki, V.M., Minion, F.C., Padula, M.P., and Djordjevic, S.P. (2013). P159 from *Mycoplasma hyopneumoniae* binds

porcine cilia and heparin and is cleaved in a manner akin to ectodomain shedding. *J Proteome Res* 12(12):5891-5903. doi: 10.1021/pr400903s.

Schmidt, J.A., Browning, G.F., and Markham, P.F. (2004). *Mycoplasma hyopneumoniae* p65 surface lipoprotein is a lipolytic enzyme with a preference for shorter-chain fatty acids. *J Bacteriol* 186(17):5790-5798. doi: 10.1128/JB.186.17.5790-5798.2004.

Seymour, L.M., Deutscher, A.T., Jenkins, C., Kuit, T.A., Falconer, L., Minion, F.C., et al. (2010). A processed multidomain *Mycoplasma hyopneumoniae* adhesin binds fibronectin, plasminogen, and swine respiratory cilia. *J Biol Chem* 285(44):33971-33978. doi: 10.1074/jbc.M110.104463.

Seymour, L.M., Falconer, L., Deutscher, A.T., Minion, F.C., Padula, M.P., Dixon, N.E., et al. (2011). Mhp107 is a member of the multifunctional adhesin family of *Mycoplasma hyopneumoniae*. *J Biol Chem* 286(12):10097-10104. doi: 10.1074/jbc.M110.208140.

Tacchi, J.L., Raymond, B.B., Jarocki, V.M., Berry, I.J., Padula, M.P., and Djordjevic, S.P. (2014). Cilium adhesin P216 (MHJ_0493) is a target of ectodomain shedding and aminopeptidase activity on the surface of *Mycoplasma hyopneumoniae*. *J Proteome Res* 13(6):2920-2930. doi: 10.1021/pr500087c.

Tao, Y., Yang, R., Shu, J., Zheng, W., Chen, J., Wu, Y., et al. (2020). Immune responses induced by a combined vaccination with a recombinant chimera of *Mycoplasma hyopneumoniae* antigens and capsid virus-like particles of porcine circovirus type 2. *BMC Vet Res* 16(1):342-354. doi: 10.1186/s12917-020-02560-8.

Vranckx, K., Maes, D., Calus, D., Villarreal, I., Pasmans, F., and Haesebrouck, F. (2011). Multiple-locus variable-number tandem-repeat analysis is a suitable tool for differentiation of *Mycoplasma hyopneumoniae* strains without cultivation. *J Clin Microbiol* 49(5):2020-2023. doi: 10.1128/jcm.00125-11.
