# Supplementary material for: Development of a Multi-Epitope Vaccine for Mycoplasma hyopneumoniae and Evaluation of Its Immune Responses in Mice and Piglets
Source: Int J Mol Sci. 2022 Jul 18;23(14):7899. doi: 10.3390/ijms23147899 (PMC9318870; doi:10.3390/ijms23147899)
Supplement: Supplementary file 1 [file ijms-23-07899-s001.zip › ijms-1768532-supplementary/Supplementary File 2.pdf]

Supplementary File S2

Table S2 Database, software and web services used in this study. [a]

Database, software or web service	Description	Uniform Resource Locator (URL) [b]
NCBI	General database	https://www.ncbi.nlm.nih.gov/
PDB	Protein structure database	https://www.rcsb.org/
BLAST	Sequence alignment	https://blast.ncbi.nlm.nih.gov/Blast.cgi
BioPerl	Perl tools for bioinformatics	https://bioperl.org/
Bio-Linux 8	Operating system for bioinformatics	http://environmentalomics.org/bio-linux-download/
Roary	Pan-genome analysis software	http://sanger-pathogens.github.io/Roary/
Psortb version 3.0	Protein subcellular localization analysis	http://www.psort.org/psortb/
CELLO2GO	Protein subcellular localization analysis	http://cello.life.nctu.edu.tw/cello2go/
Virulence Factor Database	Database for virulence factor	http://www.mgc.ac.cn/VFs/main.htm
TMHMM Server v. 2.0	Prediction of transmembrane helices in proteins	http://www.cbs.dtu.dk/services/TMHMM/
ProtParam	Protein physical and chemical parameters analysis	https://web.expasy.org/protparam/
SignalP-5.0	Protein signal peptide prediction	http://www.cbs.dtu.dk/services/SignalP/
Kolaskar and Tongaonkar method	Protein immunogenicity prediction	http://imed.med.ucm.es/Tools/antigenic.pl
ANTIGENpro	Protein immunogenicity prediction	http://scratch.proteomics.ics.uci.edu/
VaxiJen V 2.0	Protein immunogenicity prediction	http://www.ddg-pharmfac.net/vaxijen/VaxiJen/VaxiJen.html
ABCPred	B-cell epitopes prediction server	https://webs.iiitd.edu.in/raghava/abcpred/
Bcepred	Continuous B-cell epitopes prediction server	https://webs.iiitd.edu.in/raghava/bcepred/
BepiPred 2.0	Sequential B-bell epitopes predictor	http://www.cbs.dtu.dk/services/BepiPred/cite.php
EpiTOP 3.0	MHC class II binding epitopes prediction	http://www.ddg-pharmfac.net/EpiTOP3/
MHCPred V 2.0	MHC class I/II binding epitopes prediction	http://www.ddg-pharmfac.net/mhcpred/MHCPred/
VirulentPred	Bacterial virulent protein prediction	http://203.92.44.117/virulent/submit.html
IFNepitope	IFN-γ inducing prediction	https://webs.iiitd.edu.in/raghava/ifnepitope/design.php
MHC-I Binding Predictions	MHC class I binding epitopes prediction	http://tools.iedb.org/mhci/
MHC Class I Immunogenicity	MHC class I binding epitopes immunogenicity prediction	http://tools.iedb.org/immunogenicity/
RaptorX Property	Protein secondary structure property prediction service	http://raptorx.uchicago.edu/StructurePropertyPred/predict/
Robetta	Protein structure prediction service	https://robetta.bakerlab.org/submit.php
I-TASSER	Protein structure prediction service	https://zhanggroup.org/I-TASSER/
ProSA-web	Protein structure analysis	https://prosa.services.came.sbg.ac.at/prosa.php
PDBsum	Protein structure analysis	http://www.ebi.ac.uk/thornton-srv/databases/pdbsum/Generate.html
ZDOCK	Molecular docking	https://zdock.umassmed.edu/
PyMOL V1.8.2.0	Molecular visualization	https://pymol.org/2/

[a] The references were shown in the relevant positions of this paper.

[b] The URLs of some services may have changed and they can be found by keywords search on Google.

```
2019/04/30 09:57:27 Looking for 'Rscript' - found /usr/bin/Rscript
2019/04/30 09:57:27 Determined Rscript version is 3.6
2019/04/30 09:57:27 Looking for 'awk' - found /usr/bin/awk
2019/04/30 09:57:27 Looking for 'bedtools' - found /usr/bin/bedtools
2019/04/30 09:57:27 Determined bedtools version is 2.27
2019/04/30 09:57:27 Looking for 'blastp' - found /usr/bin/blastp
2019/04/30 09:57:28 Determined blastp version is 2.9.0
2019/04/30 09:57:28 Looking for 'grep' - found /usr/bin/grep
2019/04/30 09:57:28 Optional tool 'kraken' not found in your $PATH
2019/04/30 09:57:28 Optional tool 'kraken-report' not found in your $PATH
2019/04/30 09:57:28 Looking for 'mafft' - found /usr/bin/mafft
2019/04/30 09:57:28 Determined mafft version is 7.453
2019/04/30 09:57:28 Looking for 'makeblastdb' - found /usr/bin/makeblastdb
2019/04/30 09:57:28 Determined makeblastdb version is 2.9.0
2019/04/30 09:57:28 Looking for 'mcl' - found /usr/bin/mcl
2019/04/30 09:57:28 Determined mcl version is 14-137
2019/04/30 09:57:28 Looking for 'parallel' - found /usr/bin/parallel
2019/04/30 09:57:28 Determined parallel version is 20161222
2019/04/30 09:57:28 Looking for 'prank' - found /usr/bin/prank
2019/04/30 09:57:28 Determined prank version is 170427
2019/04/30 09:57:28 Looking for 'sed' - found /usr/bin/sed
2019/04/30 09:57:28 Looking for 'cd-hit' - found /usr/bin/cd-hit
Use of uninitialized value in concatenation (.) or string at /usr/share/pe
rl5/Bio/Roary/External/CheckTools.pm line 131.
2019/04/30 09:57:28 Determined cd-hit version is
Use of uninitialized value in numeric lt (<) at /usr/share/perl5/Bio/Roary
/External/CheckTools.pm line 132.
2019/04/30 09:57:28 Roary needs cd-hit 4.6 or higher. Please upgrade and t
ry again.
2019/04/30 09:57:28 Looking for 'FastTree' - found /usr/bin/FastTree
2019/04/30 09:57:28 Determined FastTree version is 2.1
2019/04/30 09:57:28 Roary version 3.13.0
```

Figure S3 Software dependency check of Roary that installed on Bio-Linux 8 system.
