# Supplementary material for: Development of a Multi-Epitope Vaccine for Mycoplasma hyopneumoniae and Evaluation of Its Immune Responses in Mice and Piglets
Source: Int J Mol Sci. 2022 Jul 18;23(14):7899. doi: 10.3390/ijms23147899 (PMC9318870; doi:10.3390/ijms23147899)
Supplement: Supplementary file 1 [file ijms-23-07899-s001.zip › ijms-1768532-supplementary/Supplementary File 4.pdf]

Supplementary File S4

The rMhp597 was expressed and purified for ELISA and antibody detection in animal experiments, this protein was reported to be a nuclease that related to cytotoxicity, inflammation and immunosuppression. The codon sequence of Mhp597 (19-377 aa, signal peptide removed) was optimized and synthesized by GenScript. The gene was inserted between the *Nco* I and *Xho* I restriction sites of pET28a, and transformed into *E.coli* Rosetta (DE3) competent cells for protein expression. The results of plasmid construction, western blotting, nickel ion affinity chromatography and nuclease identification were presented in **Figure S4**, **Figure S5**, **Figure S6** and **Figure S7**, respectively. The eluted rMhp597 was dialyzed overnight in PBS buffer with a magnetic stirrer at 4℃. The protein concentration was determined by BCA and SDS-PAGE assay, then diluted to 0.1 mg/mL and stored at -80℃. It should be noted that the transformation of the recombinant plasmid inhibited the growth rate of host and caused remarkable degradation of genome nucleic acid as presented in **Figure S8**. These results indicated that rMhp597 had been thimbleful expressed without the addition of IPTG and shown nuclease activity in *E.coli* Rosetta, while in molecular cloning host (*E.coli* TG1), the recombinant plasmid had little effect. On the other hand, the volume of each component in different reaction groups for piglet antibody activity assay were listed in **Table S3**.

Positive control	1 mol/L $MgSO_4$ (μL)	0.1 mol/L $CaCl_2$ (μL)	pUC57 (μL)	rMhp597 (μg)	ddH_2O (μL)	Total volume (μL)
1	5	5	20	0.05	19.5	
2	5	5	20	0.1	19	
3	5	5	20	0.2	18	50
4	5	5	20	0.3	15	
5	5	5	20	0.4	10	
**Negative control**	**1 mol/L $MgSO_4$ (μL)**	**0.1 mol/L $CaCl_2$ (μL)**	**pUC57 (μL)**	**PBS (μL)**	**ddH_2O (μL)**	**Total volume (μL)**
1	5	5	20	0.5	19.5	
2	5	5	20	1	19	
3	5	5	20	2	18	50
4	5	5	20	5	15	
5	5	5	20	10	10	
**Antiserum group**	**1 mol/L $MgSO_4$ (μL)**	**0.1 mol/L $CaCl_2$ (μL)**	**pUC57 (μL)**	**rMhp597 (μg)**	**antiserum (μL)**	**Total volume (μL)**
1	5	5	20	0.05	19.5	
2	5	5	20	0.1	19	
3	5	5	20	0.2	18	50
4	5	5	20	0.3	15	
5	5	5	20	0.4	10	

Table S3 Grouping and the volume of each component in the suppression assay of piglet antiserum to rMhp597 nuclease activity, 5 different scales of rMhp597 and antiserum were tested in the antiserum group, in which the dosage of rMhp597 ranged from low to high and the antiserum was the opposite.

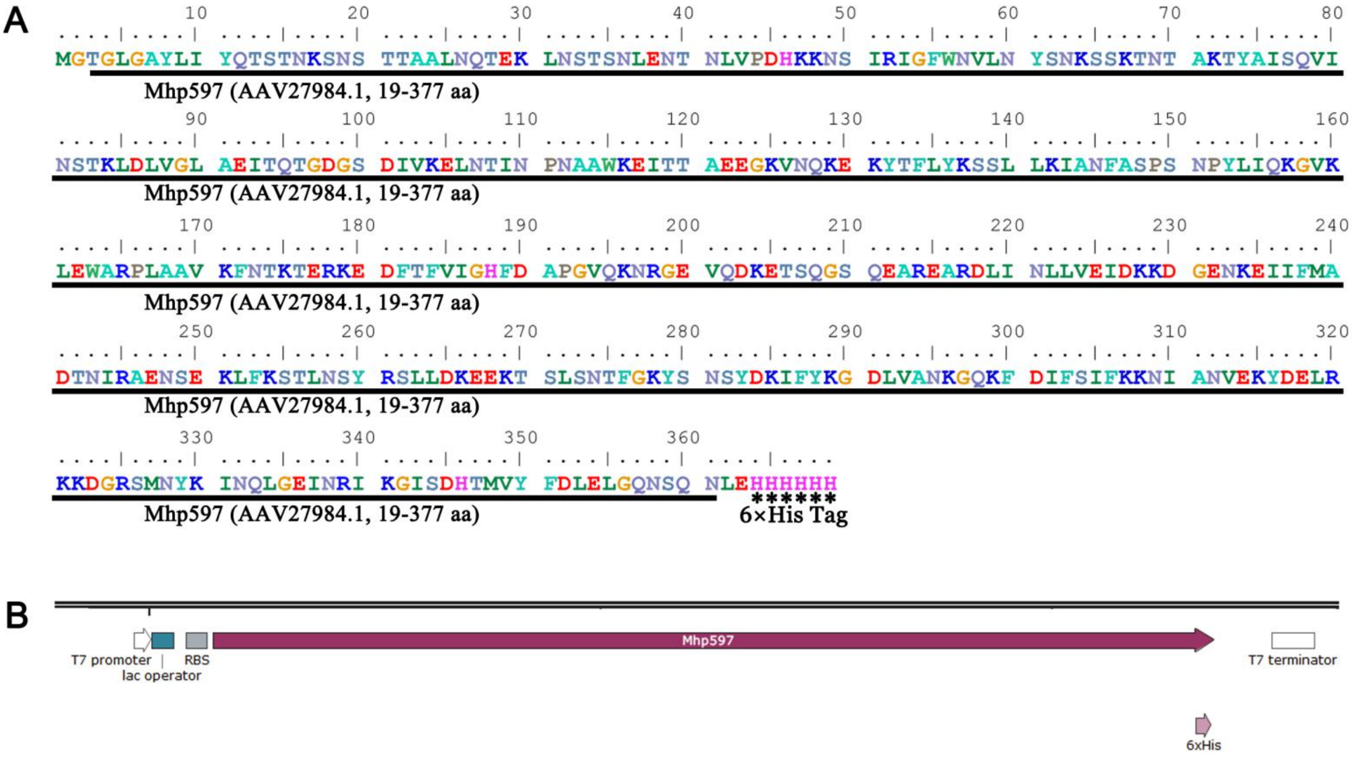

Figure S4 The protein sequence and expression vector construction of rMhp597. **(A)** The underlined sequence was from the primary protein AAV27984.1 and the 6×His Tag was indicated at the C-terminus of rMhp597. **(B)** Features of open reading framework of rMhp597 in the recombinant plasmid.

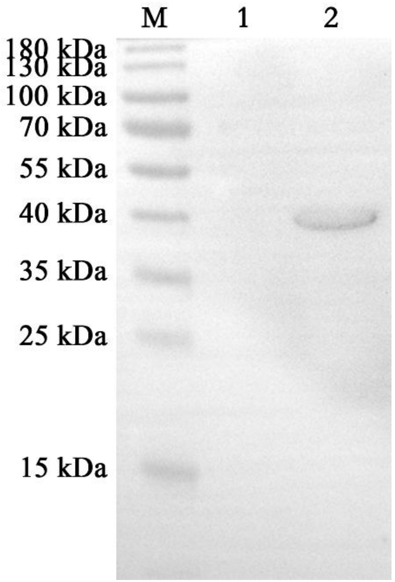

Figure S5 Western blotting analysis of recombinant protein rMhp597. After protein expression, the bacteria pellet was collected and sonicated on ice, saturated ammonium sulfate was added into the supernatant to 40% saturation. The supernatant fluid was collected 20 min later, and further added saturated ammonium sulfate to 55% saturation. The precipitate was re-suspended and dialyzed in PBS buffer, then it was loaded for SDS-PAGE and western blotting analysis. Mouse monoclonal anti-His antibody was used as the primary antibody, and the secondary antibody was HRP-labeled goat anti-mouse IgG (H+L). Lane 1: non-induced bacteria as the negative control; Lane 2: rMhp597 protein.

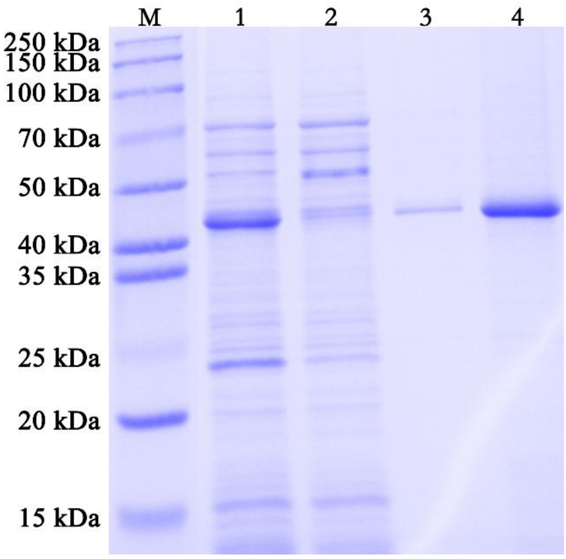

Figure S6 The nickel ion affinity chromatography purification of rMhp597. Lane 1: stock solution; Lane 2: flow-through solution. Lane 3: washing solution; Lane 4: rMhp597 elution.

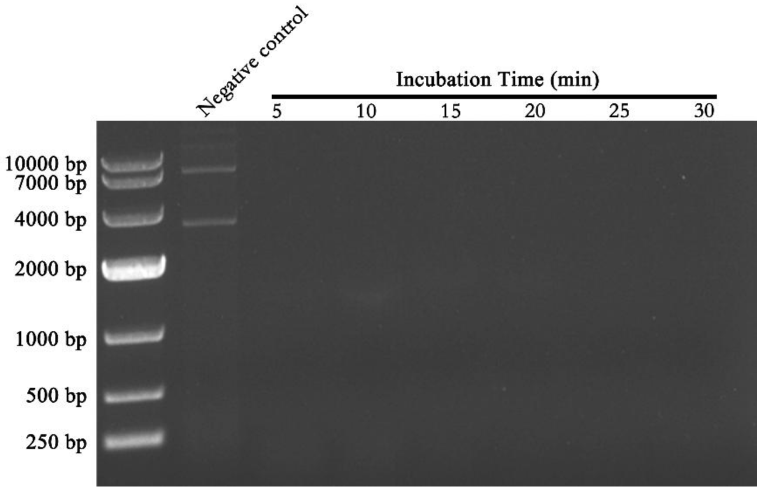

Figure S7 Identification of nuclease activity of rMhp597. The substrate for the enzymatic reaction was pET28a, Ca^{2+} and Mg^{2+} are added to the reaction system simultaneously. The mixture was incubated in a 37℃ for different times then stopped by heating in a 95℃ water bath, and the electrophoresis was performed immediately.

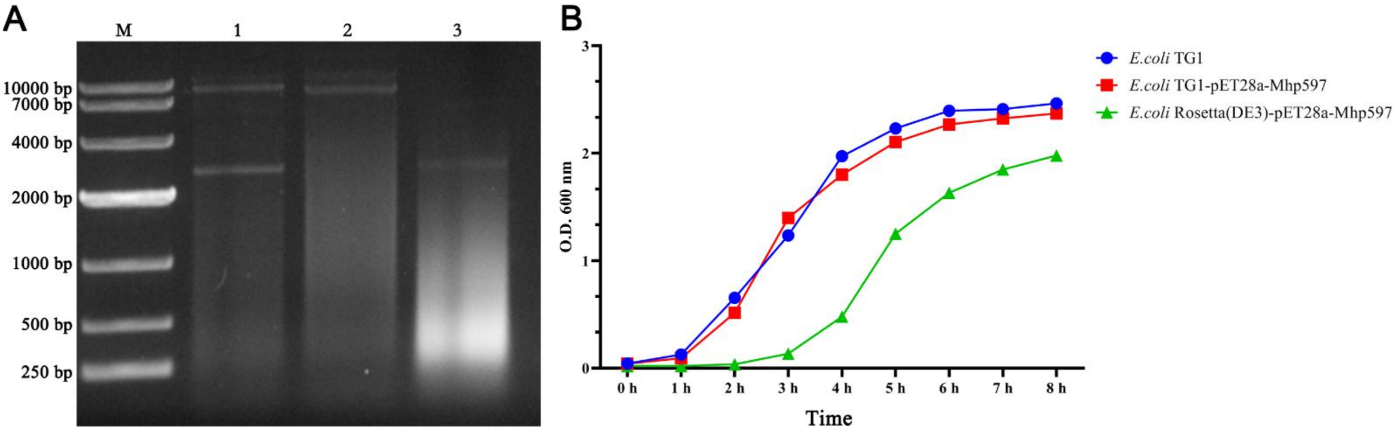

Figure S8 The impact of recombinant vector transformation on the *E.coli* host. **(A)** the nucleic acid electrophoresis results of the plasmids directly extracted from different strains, Lane 1: *E.coli* Rosetta-pET28a; Lane 2: *E.coli* TG1-pET28a-Mhp597; Lane 3: *E.coli* Rosetta-pET28a-Mhp597. **(B)** The effects of recombinant vector transformation on host growth curve.
