# Supplementary material for: Development of a Multi-Epitope Vaccine for Mycoplasma hyopneumoniae and Evaluation of Its Immune Responses in Mice and Piglets
Source: Int J Mol Sci. 2022 Jul 18;23(14):7899. doi: 10.3390/ijms23147899 (PMC9318870; doi:10.3390/ijms23147899)
Supplement: Supplementary file 1 [file ijms-23-07899-s001.zip › ijms-1768532-supplementary/Supplementary File 6.pdf]

Supplementary File S6

Sequence alignment results of the epitopes used in this study and their homologous sequences in different Mhp strains were shown in **Figure S9**. Although some epitopes had amino acid mutations, the frequency of these mutations was relatively low. MhpMEV was unable to be purified by nickel ion affinity chromatography as shown in **Figure S10**, and to further improve the purity, it was redesigned as MhpMEVC6His, the sequence alignment between MhpMEV and MhpMEVC6His was shown in **Figure S11**. The gene sequence of MhpMEVC6His was obtained from pET22b-MhpMEV plasmid with the primers listed in **Table S4**. The product was inserted between the *Bam*H I and *Eco*R I restriction sites of pET22b and transformed into *E.coli* BL21 (DE3) competent cells. The expression conditions and refolding method of MhpMEV were also used for MhpMEVC6His. MhpMEVC6His was further purified by nickel ion affinity chromatography and dialyzed overnight in 50 mM Tris-HCl, 5% glycerol, pH 8.0 to remove imidazole (**Figure S12**). The purified MhpMEVC6His was identified by 12% SDS-PAGE and stored at -80℃ until use. **Figure S13** presented the MhpMEVC6His secondary structural analysis and prediction results, which shown major differences when compared with the results of circular dichroism. The three-dimensional structure predictions and model validation results of MhpMEVC6His were shown in **Figure S14**, Robetta service could give a better model than I-TASSER and AlphaFold. The top 10 binding models for the docking results of MhpMEV and swine immune-associated receptors were listed in **Table S5**.

Primer	Sequence (5’-3’)	Product name	Product length
C6His-F-*Bam*H I	CGCGGATCCCAAACACTATACAT	MhpMEVC6His	540 bp
C6His-R-*Eco*R I	CCGGAATTCCCTCAGTGGTGGTGATG GTGATGGTCGTTAGTCTCAAA		

Table S4 Primers used for the amplification of MhpMEVC6His from plasmid pET22b-MhpMEV, the underline sequences indicated the restriction sites.

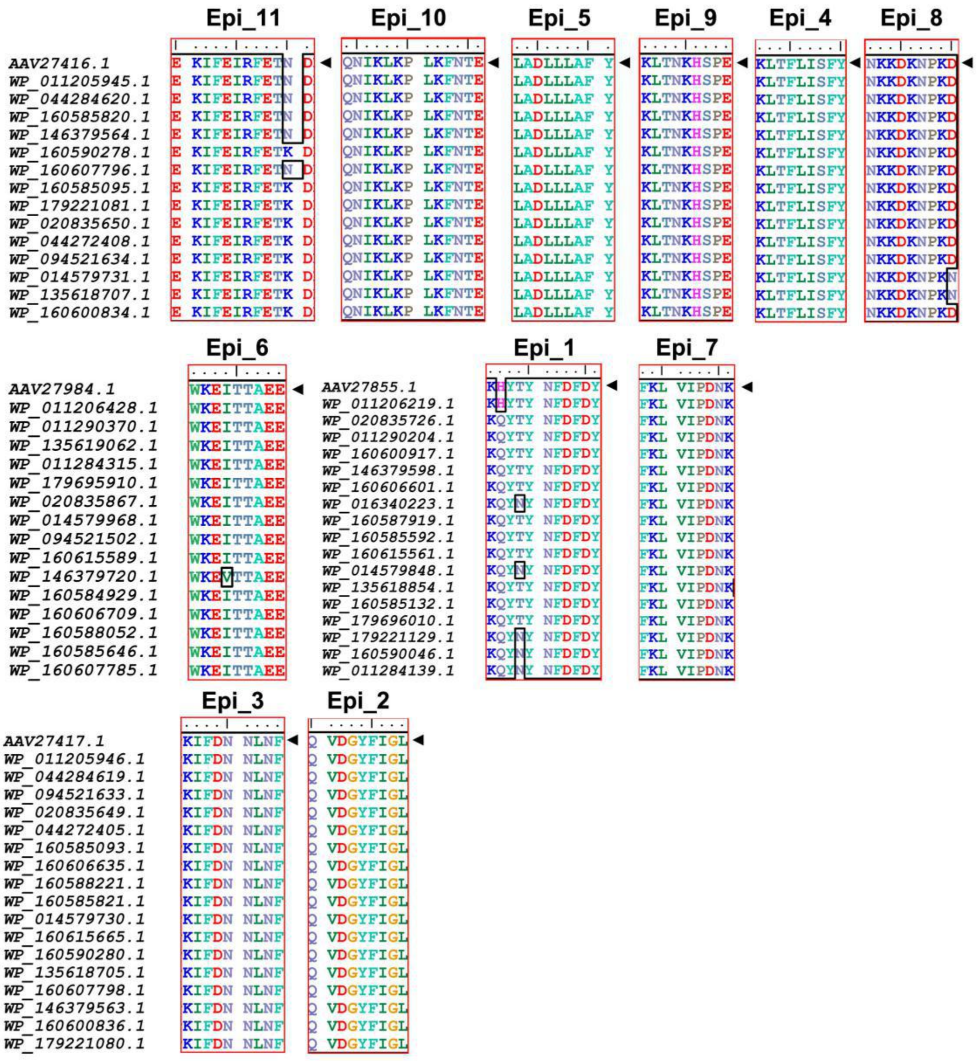

Figure S9 Sequence alignment results of the epitopes used in this study (indicated) and their homologous sequences in different Mhp strains.

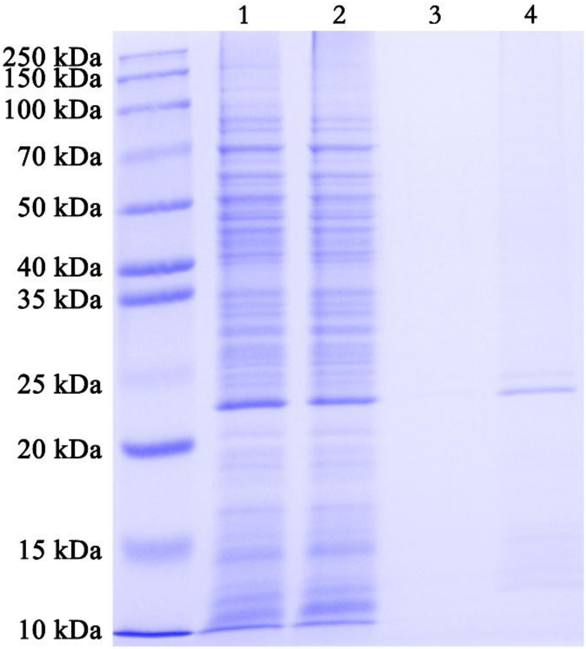

Figure S10 The nickel ion affinity chromatography purification of MhpMEV. The flow-through solution and stock solution shown no obvious difference, and indicated that the binding of MhpMEV to nickel column was ineffective. Lane 1: stock solution; Lane 2: flow-through solution. Lane 3: washing solution; Lane 4: elution.

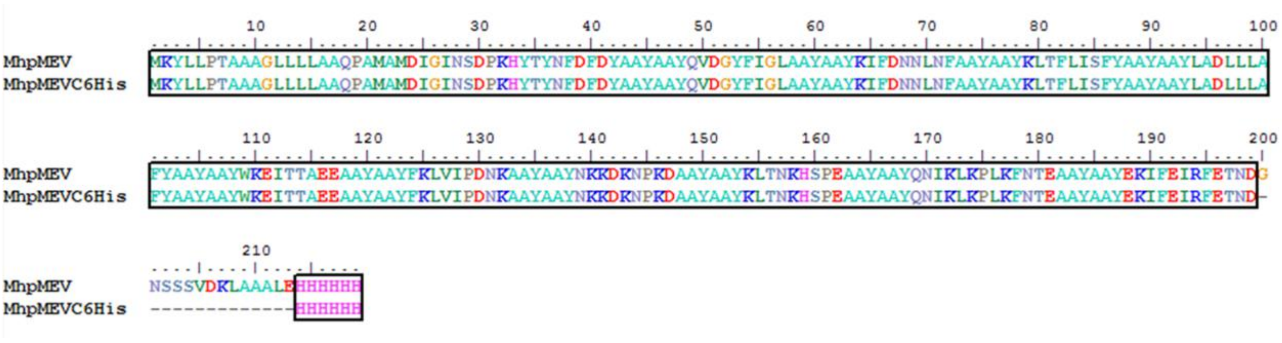

Figure S11 The sequence alignment between MhpMEV and MhpMEVC6His. MhpMEV carried the fragment that translated from plasmid sequence, which was removed in MhpMEVC6His.

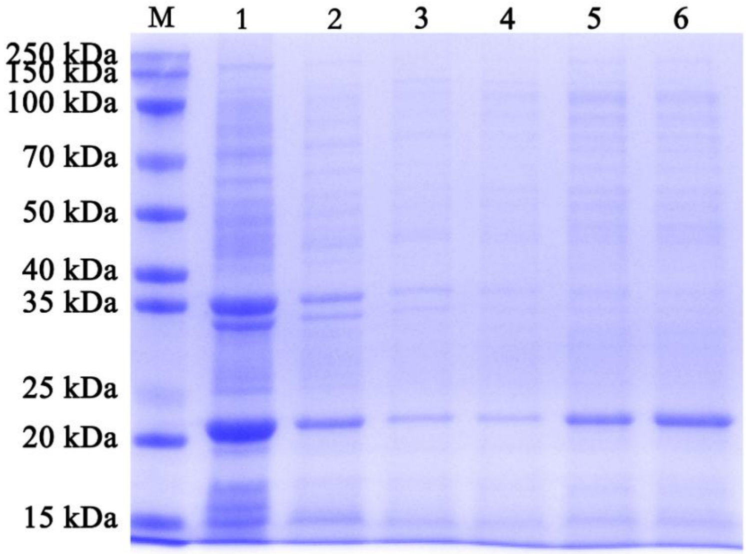

Figure S12 Purification of MhpMEVC6His by nickel ion affinity chromatography. Lane 1: stock solution; Lane 2: flow-through solution. Lane 3 and 4: washing solution; Lane 5 and 6: elution.

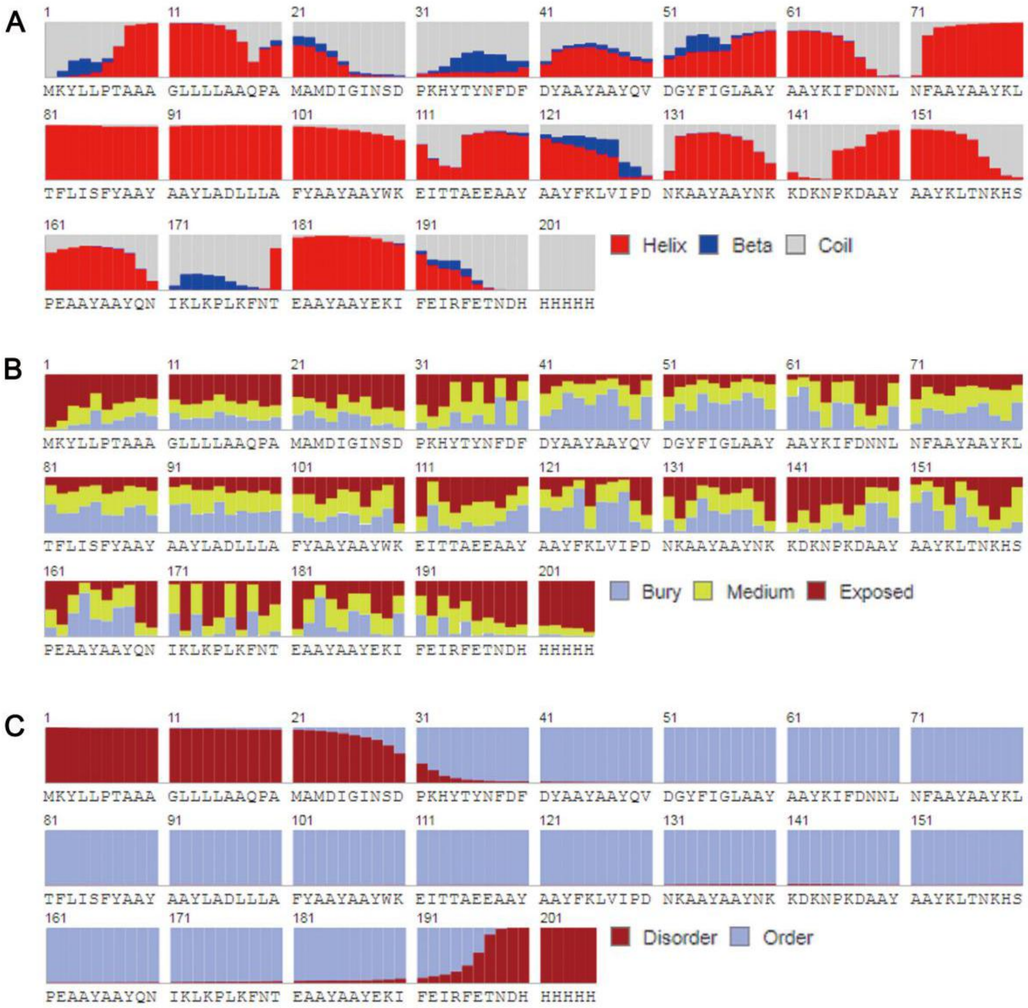

Figure S13 MhpMEVC6His secondary structural analysis and prediction results. The three-state secondary structure (alpha-helix, beta-sheet, and coil), relevant solvent accessibility (buried, medium and exposed in solvent) and disordered regions (order/disorder prediction based on the cutoff value at 0.25, respectively) calculated by RaptorX Property were shown in **(A)**, **(B)** and **(C)**, respectively.

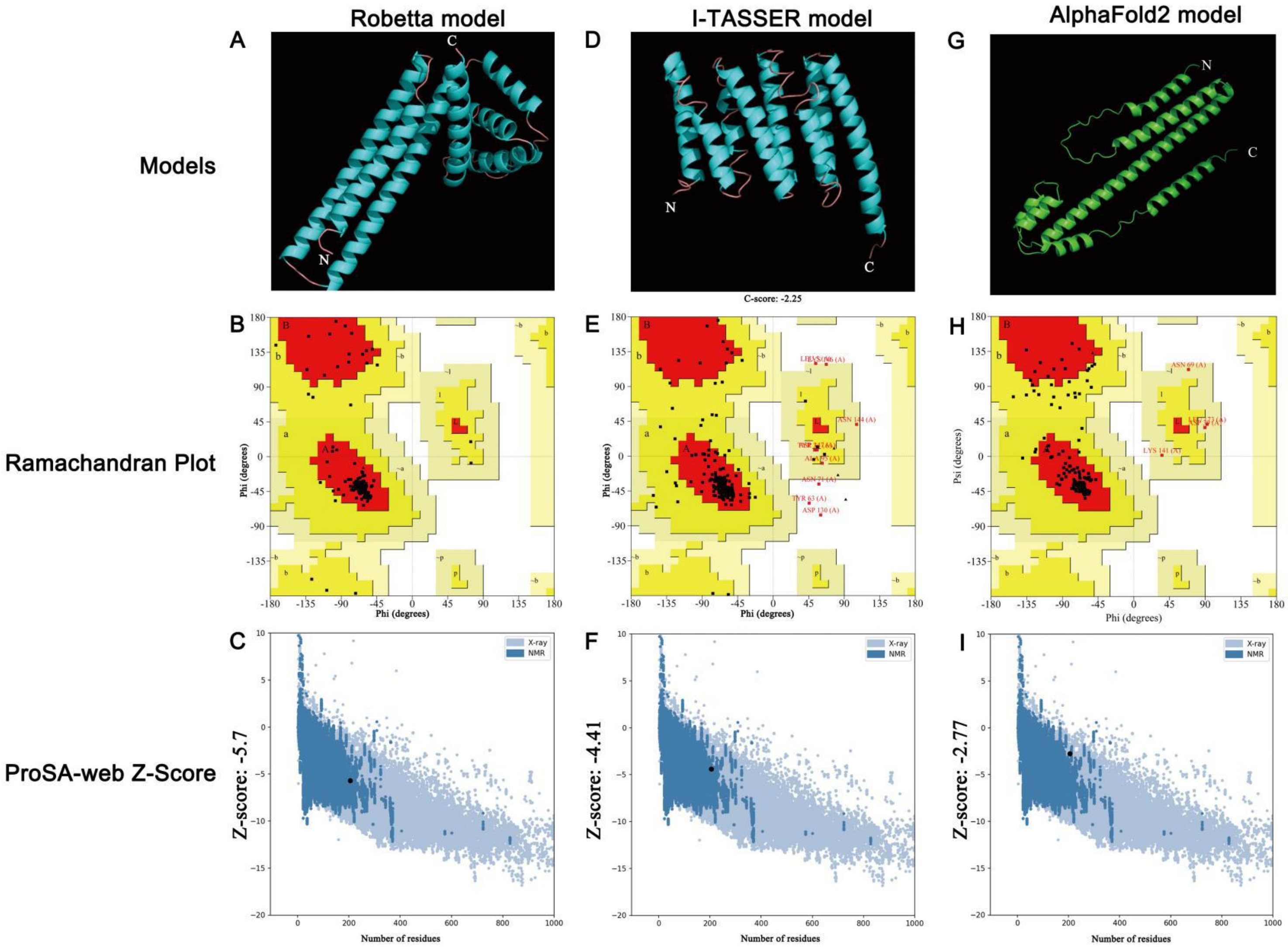

Figure S14 The molecular models of MhpMEVC6His predicted by Robetta, I-TASSER and AlphaFold were shown in **(A), (D)** and **(G)**, respectively. Ramachandran plot of the three models were shown in **(B), (E)** and **(H)** for different models. Most of the residues in Robetta model were found in favored (93.8%) or allowed (6.2%) regions, while in I-TASSER and AlphaFold2 models, 81.2% and 84.9% residues were found in favored regions, respectively. The Z-scores of the three models that calculated by ProSA Web were presented in **(C)**, **(F)** and **(I).**

Receptor	Receptor description	Euler angles (in radians) for rotating ligand			Grid positions for the translation of ligand			ZDOCK score
2A0Z	Toll-like receptor 3	-0.261799	1.982574	-1.115518	2	164	2	2122.003
		-0.523599	2.592629	0.909713	158	136	31	2083.812
		-0.261799	1.904882	-1.478089	0	167	3	2001.456
		-1.047198	2.379497	0.561089	157	137	34	1977.481
		-1.047198	1.741644	-2.695914	165	14	11	1923.951
		-1.047198	2.456751	-1.503343	165	156	164	1885.079
		-1.308997	2.115563	0.68573	146	137	30	1878.36
		-1.047198	2.29548	1.008277	145	139	32	1869.289
		0.785398	2.247462	0.191623	0	3	164	1852.68
		2.879793	1.450119	-2.73905	9	158	11	1852.607
3QQ4	MHC class I antigen	-2.617994	2.108018	1.31143	130	133	20	1891.537
		2.356194	0.914445	2.687814	138	127	12	1781.371
		2.617994	0.914445	2.687814	0	129	12	1780.73
		2.356194	0.733948	3.021411	139	126	11	1775.949
		1.570796	2.25133	-2.330739	121	115	9	1740.289
		2.356194	1.218453	2.777284	4	133	11	1714.879
		2.356194	1.41343	2.550734	135	126	17	1714.362
		2.356194	1.134796	2.43429	138	128	13	1692.432
		1.832596	2.063651	-2.0446	131	126	1	1649.754
		2.617994	1.134796	2.43429	139	128	13	1627.605
5YLX	MHC class I antigen	0.785398	0.08073	-2.622036	140	136	6	2007.297
		1.308997	0.41964	2.974122	141	136	8	1994.941
		1.570796	0.733948	3.021411	141	131	13	1964.092
		0.261799	0.08073	-2.622036	138	142	1	1817.578
		0	0.08073	-2.622036	138	139	6	1802.457
		1.047198	0.41964	2.974122	141	138	5	1794
		-1.308997	1.184361	0.625864	7	126	2	1756.124
		-0.785398	0.961715	0.014085	0	133	143	1741.879
		-1.308997	0.850607	-1.335383	136	134	5	1714.93
		-1.047198	1.045978	0.343692	4	127	3	1709.812
6A6H	MHC class I antigen	-2.356194	1.448123	-2.163292	4	13	14	2083.16
		-2.094395	1.374601	-2.456031	2	14	16	2036.655
		-2.356194	1.545873	-1.877082	3	10	11	1889.559
		-2.617994	1.214854	-1.966664	179	15	15	1887.551
		1.308997	2.664082	-1.071402	169	1	146	1877.025
		1.047198	1.450119	-2.73905	164	177	20	1764.946
		-1.832596	1.448123	-2.163292	176	16	15	1754.035
		1.570796	2.664082	-1.071402	168	179	147	1745.854
		-2.094395	1.448123	-2.163292	175	15	15	1709.898
		2.094395	2.115563	0.68573	1	14	21	1686.438
7EMA	Leucocyte antigen	1.832596	2.174483	2.653697	13	108	0	1654.033
		-1.308997	1.998705	2.370472	134	0	15	1634.793
		-2.356194	2.704512	1.488683	130	0	7	1633.986
		-1.832596	2.393226	1.857173	129	141	10	1589.832
		-2.094395	2.393226	1.857173	130	143	11	1580.318
		-1.832596	2.174483	2.653697	134	4	10	1498.435
		-0.523599	2.115563	0.68573	4	105	139	1496.288
		-1.570796	2.637879	2.121598	130	0	7	1486.805
		2.617994	1.766067	2.153843	136	131	28	1483.921
		-1.832596	2.299159	2.264663	130	143	11	1482.314

Table S5 Molecular docking results of MhpMEVC6His with the following swine immune-associated receptors included 2A0Z, 3QQ4, 5YLX, 6A6H and 7EMA. The docked complex was generated by ZDOCK service, and the top 10 predictions with the highest ZDOCK scores were listed.
